# Genome-wide identification and comparative expression profiling of the WRKY transcription factor family in two *Citrus* species with different *Candidatus* Liberibacter asiaticus susceptibility

**DOI:** 10.1186/s12870-023-04156-4

**Published:** 2023-03-24

**Authors:** Wen-Shan Dai, Ting Peng, Min Wang, Ji-Hong Liu

**Affiliations:** 1 College of Life Sciences, National Navel Orange Engineering Research Center, Gannan Normal University, Ganzhou, Jiangxi 341000 China; 2 College of Horticulture and Forestry Sciences, National Key Laboratory for Germplasm Innovation & Utilization of Horticultural Crops, Huazhong Agricultural University, Wuhan, Hubei 430070 China

**Keywords:** Citrus, WRKY transcription factor, Genome-wide identification, Salicylic acid, Huanglongbing

## Abstract

**Background:**

Salicylic Acid (SA) is a pivotal phytohormone in plant innate immunity enhancement of triggered by various pathogens, such as *Candidatus* Liberibacter asiaticus (*C*Las), the causal agent of Huanglongbing (HLB). WRKY is a plant specific transcription factor (TF) family, which plays crucial roles in plant response to biotic stresses. So far, the evolutionary history, functions, and expression patterns under SA treatment and *C*Las infection of WRKY family are poorly understood in *Citrus*, despite the release of the genome of several *Citrus* species. A comprehensive genomic and expressional analysis is worth to conduct for this family.

**Results:**

Here, a genome-wide identification of WRKY TFs was performed in two *Citrus* species: *Citrus sinensis* (HLB-sensitive) and *Poncirus trifoliata* (HLB-tolerant). In total, 52 *CsWRKYs* and 51 *PtrWRKYs* were identified, whose physical and chemical properties, chromosome locations, phylogenetic relationships and structural characteristics were comparatively analyzed. Especially, expression patterns of these *WRKY* genes before and after SA treatment and *C*Las infection were compared. Based on this result, seven pairs of orthologous *WRKY* genes showing opposite expression patterns in two *Citrus* species were screened out. Moreover, two pairs of orthologous *WRKY* genes with significant differences in the number or type of stress-responsive *cis*-elements in the promoter regions were discovered. Subcellular localization and transcriptional activation activity assays revealed that these two pairs of orthologous genes are classic WRKY TFs localize in the nucleus and could function as transcriptional activators.

**Conclusion:**

In this study, we systematically analyzed the genomic characterization of *WRKY* family in two *Citrus* species, together with the analyses of expression patterns under SA signaling and *C*Las infection. Our study laid a foundation for further study on the function of WRKY TFs in HLB response and SA signaling of *Citrus*.

**Supplementary Information:**

The online version contains supplementary material available at 10.1186/s12870-023-04156-4.

## Background

Huanglongbing, generally referred to as citrus greening, is the most catastrophic and unprecedented disease that impedes citrus growth and threatens global citrus industry [1]. Three ‘*Candidatus* Liberibacter’ species, ‘*Candidatus* Liberibacter asiaticus’, ‘*Candidatus* Liberibacter americanus’, and ‘*Candidatus* Liberibacter africanus’, have been identified as the causal agents of HLB, among which *C*Las is the most widespread and harmful species [2]. Two phloem-feeding citrus psyllids, *Diaphorina citri* (Asian citrus psyllid, ACP) and *Trioza erytreae*, are natural vectors responsible for the transmission of HLB-associated bacteria [3]. Once infected with *C*Las, citrus trees will exhibit a series of typical symptoms including yellow or green mottled spots in leaves, stunted twigs, lopsided fruits and abortive seeds. Eventually, the trees will die as the disease progresses [4].

To date, there is no radical cure to eliminate HLB disease. Almost all citrus cultivars can be affected by the HLB-associated bacteria and there is no confirmed resistance [5]. However, the preliminary analysis of different citrus cultivars and varieties has indicated that there are differences in host response to *C*Las [6]. Evidences showed that many trifoliate orange varieties and its hybrids exhibited lighter symptoms of HLB than the rootstock shoots emerging from the trees’ bases did [7–9]. Several accessions of *P. trifoliata* have also been found to be resistant to ACP by influencing oviposition and lifespan of adults [10, 11]. Therefore, trifoliate orange may have a certain tolerance to *C*Las infection or psyllid colonization.

SA is a ubiquitous phenolic compound distributed extensively in plants and a vital phytohormone that regulates various stress responses and physiological processes, such as immune response, seed germination, growth and development, stomatal closure, senescence, etc. [12]. Among them, the function of SA in plant immune response to pathogens has been extensively studied. SA signaling constitutes a tightly linked regulatory cascade that plays a pivotal role in establishing Systemic Acquired Resistance (SAR), a long-term and broad-spectrum resistance which exists throughout the whole plant after local pathogen infection [13]. SA content in plants elevates rapidly after pathogen infection, which could be transported to various tissues and organs, and finally induce a series of SAR resistance reactions, such as the expression of disease resistance genes, the interaction between SA and disease resistance proteins, and between other related genes through signal transduction. Recent studies have demonstrated that SA resistance signaling pathway is a complex network, in which multiple TFs are involved to regulate gene expression after pathogen infestation [14, 15].

WRKY TFs comprised plenty members in plant genomes and form indispensable parts of regulator networks that modulate numerous plant processes [16–19]. The name of the WRKY TFs is derived from the most conspicuous characteristic of these proteins, namely the WRKY domain, a highly conserved region containing approximately 60 amino acids with a core sequence WRKYGQK at its N-terminal end, together with a novel zinc-finger-like domain at its C-terminal [20]. The WRKY proteins are generally categorized into three groups and eight subgroups on the basis of the number of WRKY domain and the feature of zinc finger motif. The proteins possessing two WRKY domains at N-terminal (Group Ia) or C-terminal (Group Ib) were classified into Group I, while those with only one WRKY domain belonged to Group II or Group III. WRKY TFs bind to the *cis*-element W-box [(T/C)TGAC(T/C)] in the promoter region of its target genes and regulate the expression of related genes [21]. Massive studies have shown that WRKY TFs participate in various biological and physiological processes, including disease resistance [22, 23]. A subgroup IIb WRKY family member CaWRKY6 from *Capsicum annuum* positively function in *Ralstonia solanacearum* resistance by activating another *WRKY* gene, *CaWRKY40* [24]. In rice, OsWRKY62 functions as a negative regulator of innate immunity by affecting both basal and *Xa21*-mediated rice-specific defense responses to the bacterial pathogen *Xanthomonas oryzae* pv. *oryzae* [25]. In summary, WRKY family has numerous members with diverse quantitative distributions in different plants and variable mechanisms under pathogen attack.

Although several WRKYs functioning in plant disease defense have been characterized, the molecular mechanisms and the regulatory roles of most WRKYs, especially those from the non-model plants, still remain poorly understood. In the current study, a genome-wide analysis of WRKY TFs was performed in two *Citrus* species, *C*. *sinensis* and *P*. *trifoliata*, by implementing bioinformatics approach on the publicly available database of sequenced genome. Multiple sequence alignments, phylogenetic relationships, chromosome distributions, gene duplications, syntenic relationships of *WRKY* genes were performed. Furthermore, the expression profiles of *WRKY* orthologous pairs from two *Citrus* species under SA treatment and *C*Las-infection were analyzed. Promoter analysis was carried out to investigate stress-responsive *cis*-elements in seven pairs of orthologous *WRKY* genes which exhibited strong opposite expression patterns. Additionally, subcellular localization and transcriptional activation activity of two pairs of orthologous *WRKY* genes with different promoter elements were also investigated. This study provides prospective knowledge for the properties and biological significance of the WRKY family in *Citrus* and functional research involved in HLB response.

## Methods

### Plant materials and growth conditions

Two *Citrus* varieties, sweet orange (*C*. *sinensis*) and trifoliate orange (*P*. *trifoliata*), were grown in containers in Gannan Normal University, Ganzhou, China. Three-month-old plants of each variety were kept under 16: 8 h, light: dark conditions at a temperature of 25 °C. Solutions of 2 mM SA were used to irrigate the potting soil and spray the branches and leaves. Each pot applied the same dosage. Leaves were collected at designated time points, instantly frozen in liquid nitrogen and stored at -80 °C for analyses of gene expression. Furthermore, leaves of *C*. *sinensis* and *P*. *trifoliata* were also collected for the RNA extraction and the preparation of cDNA template, which was further used for gene cloning and vector construction. Tobacco (*Nicotiana benthamiana*) plants were planted on potted soil under the same growth conditions for subsequent transient transformation experiment. Three leaves, sampled randomly from three different pots (one leaf per pot), were used as repetitions for each condition.

Two-year-old plants of each citrus varieties were graft-inoculated with axillary bud of HLB-symptomatic sweet orange plants, which were grafted on the plant’s primary stem. At least three axillary buds were grafted for each plant. Axillary buds from healthy plants were utilized to graft two citrus varieties as control. All plants were maintained in a greenhouse with an average temperature of 25 °C. Leaves and petioles were randomly collected at three months after inoculation for DNA isolation and RNA extraction, with three biological replicates for each experiment.

#### Screening and identification of the WRKY genes in C. sinensis and P. trifoliata

The WRKY protein sequences of *C*. *sinensis* and *P*. *trifoliata* were obtained from Citrus Pan-genome to Breeding Database (http://citrus.hzau.edu.cn/index.php), respectively. The Hidden Markov Model (HMM) of the WRKY domain (PF03106) was downloaded from the Pfam protein family database (http://pfam.xfam.org/) [26] and used to identify putative WRKY proteins using the HMMER search program (http://hmmer.janelia.org/, Version 3.0) with an E-value of less than 1e^− 5^. All non-redundant protein sequences were selected as putative WRKY proteins and confirmed the presence of the complete WRKY domains using the SMART software program (http://smart.embl-heidelberg.de/) [27] and the CDD software program (http://www.ncbi.nlm.nih.gov/Structure/cdd/wrpsb.cgi) [28]. Genes without the conserved WRKY domains were excluded, and only the longest transcript could be reserved. The relative molecular weight (MW) and isoelectric point (pI) of the WRKY proteins from both varieties were calculated using the Compute pI/MW tool ExPASy (https://web.expasy.org/compute_pi/) [29].

#### Phylogenetic, gene structure and motif analysis

Multiple sequence alignment of WRKY proteins was performed through the ClustalW (https://www.genome.jp/tools-bin/clustalw) for each species individually. Phylogenetic analysis of WRKY peptides based on amino acid sequences was performed separately for three species (*C*. *sinensis*, *P*. *trifoliata* and *A*. *thaliana*) following Neighbor-joining method in MEGA (https://www.megasoftware.net/, Version 11.0), with the maximum likelihood method (bootstrap: 1,000 replicates) [30]. The protein sequences of AtWRKYs were obtained from The Arabidopsis Information Resource (TAIR) database (https://www.arabidopsis.org/). The Gene Structure Display Server program (GSDS, http://gsds.gao-lab.org/index.php, Version 2.0) was used to illustrate the exon-intron structures through matching the genomic sequences and coding sequences (CDS) of the obtained *WRKY* genes [31]. MEME program (http://meme-suite.org/tools/meme, Version 5.4.1) was employed to predict the conserved motifs in different WRKY proteins [32]. The analysis was performed by keeping number of repetitions, any; maximum number of motifs, 10; and optimum width of the motif ≥ 20.

#### Chromosomal location and gene duplication analysis

Precise position information on chromosomes for the genes encoding these CsWRKY and PtrWRKY proteins were obtained from the Citrus Pan-genome to Breeding Database. Chromosome location map of the *WRKY* genes was generated by the software MapInspect (http://mapinspect.software.informer.com/). The genes were plotted separately onto the nine chromosomes according to their ascending order of physical position (bp), from the short-arm telomere to the long-arm telomere. To detect segmental and tandem duplication events, every *WRKY* sequence was respectively aligned against the other WRKY protein sequences in *C*. *sinensis* or *P*. *trifoliata* respectively. BLASTp program was used to identify potential homologous gene pairs (E-value < 1e^− 5^, top three matches) and output format as tabular. Subsequently, the destination tabular file and the GFF file of *C*. *sinensis* or *P*. *trifoliata* genome were inputted into software MCScanX to analyze duplication types with the flowing parameters: match score (> 20); gap penalty (-1); match size (5); E-value: 1e^− 5^; max gaps (25) and visualized using software program CIRCOS (http://circos.ca/) [33].

#### DNA isolation and *C*Las quantification

Young leaves of graft-inoculated *C*. *sinensis* and *P*. *trifoliata* plants with similar age and position were collected. Petioles of three leaves from each plant were combined and grind into powder. DNA extraction was carried out using the CTAB (cetyltrimethylammonium bromide) method according to prior method [34]. DNA concentration was determined using a NanoDrop 2000 (NanoDrop Technologies, Wilmington, DE, USA) and adjusted to 20 ng/µL. Three biological replicates were randomly sampled for each variety sample.

The presence of the *C*Las pathogen in the isolated DNA samples were confirmed by PCR using the *C*Las *16 S* F/*C*Las *16 S* R primers, and the degree of *C*Las infection was determined by quantitative PCR (qPCR) according to the protocol of prior research [35]. The *C*Las *16 S* gene was amplified using q*C*Las *16 S* F/q*C*Las *16 S* R primers and citrus *18 S* gene was detected as the internal reference using qCt *18 S* F /qCt *18 S* R primers, respectively (Supplementary Table S1). Each sample was analyzed in three biological replicates and three technical replications. Referring to the Ct values of the *C*Las *16 S* gene, the tested samples with Ct < 31.3 were identified as positive for *C*Las, and samples with Ct > 36 were *C*Las negative. The *C*Las bacterial populations (*C*Las cells µg^− 1^ of citrus DNA) were calculated using the formula described by prior study [36]. Samples with similar *C*Las bacterial populations were selected for further gene expression analysis.

#### RNA extraction and quantitative real-time (qRT)-PCR analysis

Total RNA was extracted from leaves in accordance with the manufacturer’s instructions of the RNeasy® Plant Mini kit (Qiagen, Germany). The quality and integrity of the total RNA were examined with agarose gel electrophoresis and the NanoDrop 2000. The cDNA was synthesized using PrimeScript™ RT reagent Kit with gDNA Eraser (Takara, Japan). The specific *WRKY* gene primers were designed using Primer Premier 5.0 software (Supplementary Table S1). Realtime qRT-PCR analysis was done using SYBR GREEN PCR Master Mix (TaKaRa, Japan) on a QuantStudio 5 Applied BioSystem (ThermoFisher Scientific, USA). To normalize the relative expression level of the target genes, *Actin* was used as the internal reference gene. The qRT-PCR reactions were repeated in three biological and three technical replications, and the 2^−ΔΔCt^ method was applied to calculate the relative expression levels. The heatmaps were constructed using the TBtools software (Version 1.098696) [37], based on the transformed data of log_2_ values. If an expression level of *WRKY* gene was more than 2 folds or less than 0.5 fold, we considered the *WRKY* gene as a significantly expressed gene. The qRT-PCR assays were performed according to prior method [38]. Venn diagram were generated using TBtools software (Version 1.098696) [37], to depicts number of differentially expressed genes commonly found among the two treatments (SA or *C*Las infection) in *C*. *sinensis* or *P*. *trifoliata*, separately.

#### Promoter analysis of putative stress-responsive *CsWRKYs* and *PtrWRKYs*

The 2.0 kb upstream promoter sequences from the transcription start site of the selected *WRKY* genes were retrieved using the ‘Sequence Fetch’ tool of Citrus Pan-genome to Breeding Database in order to perform promoter analysis. Two plant *cis*-elements database, PlantCARE (http://bioinformatics.psb.ugent.be/webtools/plantcare/html/) and New PLACE (https://www.dna.affrc.go.jp/PLACE/?action=newplace), were used to analyze the stress-response elements in promoters [39, 40]. The identified *cis*-elements were then visualized by TBtools (Version 1.098696) [37].

#### Subcellular localization assay

The full length (FL) of coding sequences without stop codon of *CsWRKY7*, *PtrWRKY39*, *CsWRKY33* and *PtrWRKY32*, were separately cloned and inserted into the pBI121-EGFP vector at *Xba* I and *Xma* I restriction sites, under the control of the Cauliflower mosaic virus 35 S (CaMV 35 S) promoter. The fusion plasmids and the empty vector were then transformed into *Agrobacterium tumefaciens* (GV3101), and the suspensions of *Agrobacteria* were co-transformed with a plasmid coding for a nuclear marker gene VirD2NLS fused to mCherry into tobacco (*Nicotiana benthamiana*) leaves by *Agrobacterium tumefaciens* infiltration. The green fluorescence (for GFP) and red fluorescence (for mCherry) were observed through a confocal laser scanning microscope (Leica TCS SP8, Germany) after infected for three days. Transformation and infiltration of tobacco were carried out according to prior study [41].

#### Transactivation assay

The FL or three truncated fragments (N, W, and C) of four *WRKY* genes (*CsWRKY7*, *PtrWRKY39*, *CsWRKY33* and *PtrWRKY32*) were amplified and inserted into pGBKT7 vector at *Eco*R I and *Bam*H I restriction sites to generate sixteen constructs. The recombinant plasmids and the negative control, pGBKT7 plasmids, were further separately transformed into Y2HGold yeast cells according to the manufacturer’s protocol (Matchmaker® Gold Yeast Two-Hybrid Library Screening System, Takara). The SD/-Trp medium were used to cultivate the yeast transformants. Transcriptional activation activity of the transformed yeast cells was monitored after incubation at 30℃ for three to five days on SD/-Trp or SD/-Trp/-His/-Ade medium supplemented with the chromogenic substrate X-α-gal (Sigma-Aldrich, USA).

## Results

### Identification of WRKY TFs in ***C. sinensis*** and ***P. trifoliata***

To identify WRKY family genes in *C*. *sinensis* and *P*. *trifoliata*, Hidden Markov Model (HMM) profile from the Pfam database and BLASTp search were performed against reference genomes using the consensus sequence of WRKY domain. In the genome assemblies of *C*. *sinensis* and *P*. *trifoliata*, 94 *CsWRKYs* and 87 *PtrWRKYs* were identified. After removing the redundant sequences and the sequences with only partial WRKY domains, 52 *WRKY* members were identified in *C*. *sinensis* and 51 were identified in *P*. *trifoliata*. Detailed information of *CrWRKYs* and *PtrWRKYs* were presented in Supplementary Table S2 and S3 respectively, including gene ID, chromosome No., strand information, start and end position on chromosome, exon and intron number, length of Open Reading Frame (ORF) sequence, amino acid (aa) number, relative molecular weight, isoelectric point and classification of individual gene members. The identified WRKYs were named according to their ascending orders on the nine chromosomes.

In *C*. *sinensis*, the lengths of the CsWRKY proteins ranged from 116 (CsWRKY2) to 724 aa (CsWRKY14), with an average of approximately 381 aa. The molecular weights of the CsWRKY proteins were between 13.35 kDa (CsWRKY2) and 78.03 kDa (CsWRKY14). The predicted isoelectric point values of CsWRKYs varied from 5.06 (CsWRKY41) to 9.83 (CsWRKY37). Out of 52 members, 22 were identified on the positive strand and 30 were on the negative strand (Supplementary Table S2).

The identified *PtrWRKY* encode peptides, which ranged from 162 (PtrWRKY8/9) to 1390 aa (PtrWRKY21) with an average length of 428 aa. The molecular weight and isoelectric point of PtrWRKY peptides were ranging from 18.22 (PtrWRKY34) to 159.05 kDa (PtrWRKY21) and 4.94 (PtrWRKY50) to 9.80 (PtrWRKY51) respectively. Out of 51 members, 26 were identified on the negative strand and 25 on the positive strand (Supplementary Table S3).

### Multiple sequence alignment and phylogenetic analysis

**Fig. 1:**
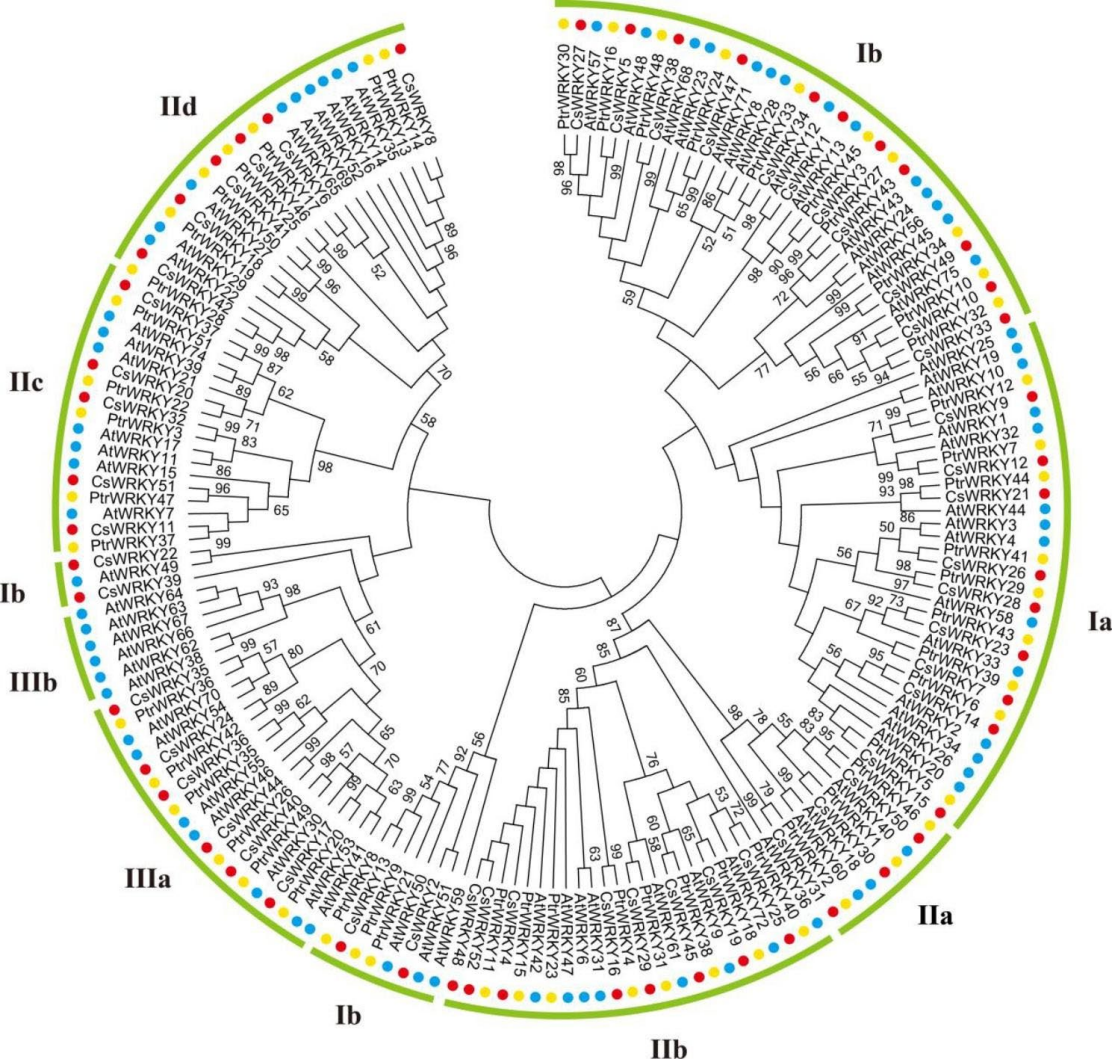
Phylogenetic analysis of WRKYs in *Citrus sinensis* (Cs), *Poncirus trifoliata* (Ptr) and *Arabidopsis thaliana* (At). The phylogenetic tree was created using MEGA by the Neighbor-Joining (NJ) method with 1,000 bootstrap replicates. The CsWRKYs (red), PtrWRKYs (yellow) and AtWRKYs (blue) were clustered into groups I, II, and III including eight subgroups (Ia; Ib, IIa, IIb, IIc, IId, IIIa, IIIb)

In order to further classify the WRKY proteins, we analyzed multiple sequence alignment of domain sequences, and constructed a NJ phylogenetic tree of CsWRKY (52 members) and PtrWRKY proteins (51 members) using WRKY sequences of model plants *Arabidopsis thaliana* (72 members) [42] as reference to represent each group and subgroup filled with different colors (Fig. 1).

Generally, the WRKY proteins are classified in three groups and five subgroups, depending on the number of the WRKY domain and the feature of zinc finger motif they possess. The members having two WRKY domains at N-terminal (Group Ia) and C-terminal (Group Ib), along with a C2H2 type zinc-finger motif, were grouped into Group I, while the members possessing only one WRKY domain and a C2H2 type zinc-finger motif belonged to Group II, and Group III members have only one WRKY domain and a C2H type zinc-finger motifs. The WRKY domain contains one or two highly conserved short peptide WRKYGQK, which considered to be important for recognizing and binding to W-box elements. A multiple sequence alignment of the core WRKY domain of CsWRKYs and PtrWRKYs was shown in Supplementary Figure S1. WRKYGQK sequences represented the major variant in 52 WRKY proteins from *C*. *sinensis* and 51 WRKY proteins from *P*. *trifoliata*. The detailed study showed that the variation arose from amino acid substitutions of Q amino acid to K amino acid, and the mutated WRKYGKK sequence was observed only in five WRKY proteins (CsWRKY2, CsWRKY13, PtrWRKY8, PtrWRKY9 and PtrWRKY21). Interestingly, the two CsWRKY proteins and three PtrWRKY proteins carrying these mutations occur in the same Group Ib.

**Fig. 2:**
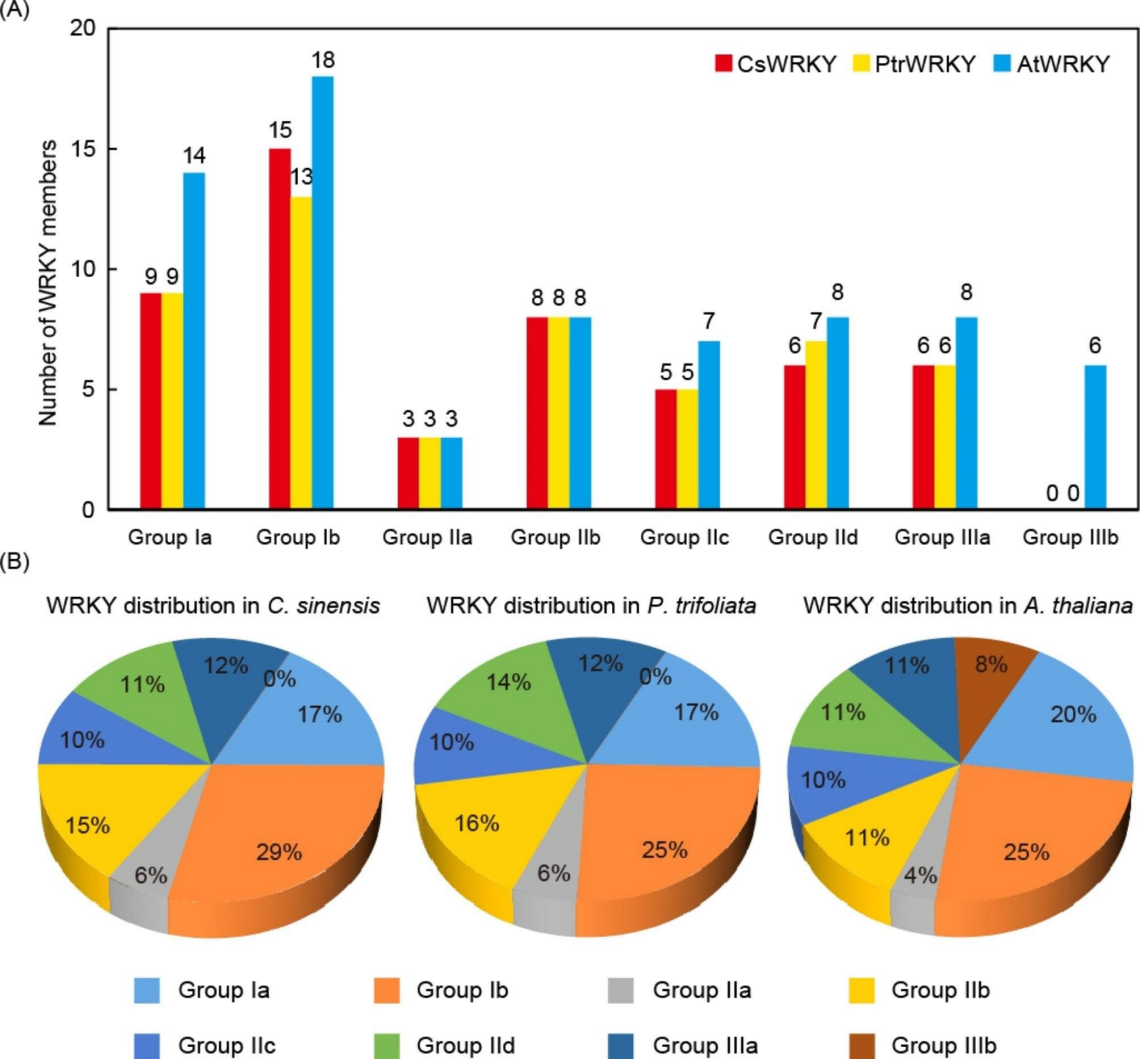
Distribution of *WRKY* genes in different species and groups. **(A)** Number of *WRKY* members in each subgroup of *Citrus sinensis*, *Poncirus trifoliata* and *Arabidopsis thaliana*. **(B)** Percentage distribution of *WRKY* members in *C*. *sinensis*, *P*. *trifoliata* and *A*. *thaliana*

As per our research findings, the CsWRKY protein family have 9 members in Group Ia, 15 in Group Ib; 3 in Group IIa, 8 in Group IIb, 5 in Group IIc, 6 in Group IId; and 6 in Group IIIa. While in *P. trifoliata*, we found 9 genes in Group Ia, 13 in Group Ib, 3 in Group IIa, 8 in Group IIb, 5 in Group IIc, 7 in Group IId and 6 encoding PtrWRKY proteins in Group IIIa. Noteworthily, neither *C*. *sinensis* nor *P*. *trifoliata* has any Group IIIb members (Fig. 2A). The distribution of *WRKY* genes in *C*. *sinensis* was comparable to that of *P*. *trifoliata* (Fig. 2B). The number of WRKY members of Group Ia, Group IIa, Group IIb, Group IIc and Group IIIa in *C*. *sinensis* and *P*. *trifoliata* were exactly the same, and their distributions in these two *Citrus* species were nearly same, namely 17.3% (Ia in *C*. *sinensis*) and 17.4% (Ia in *P*. *trifoliata*), 5.8% and 5.9% (IIa), 15.4% and 15.7% (IIb), 9.6% and 9.8% (IIc) and 11.5% and 11.8% (IIIa), respectively. Although the numbers of WRKY members differ in most subgroups between *Citrus* and *Arabidopsis*, the distribution of WRKYs was similar among these crops.

#### Gene structure analysis of WRKY genes

**Fig. 3:**
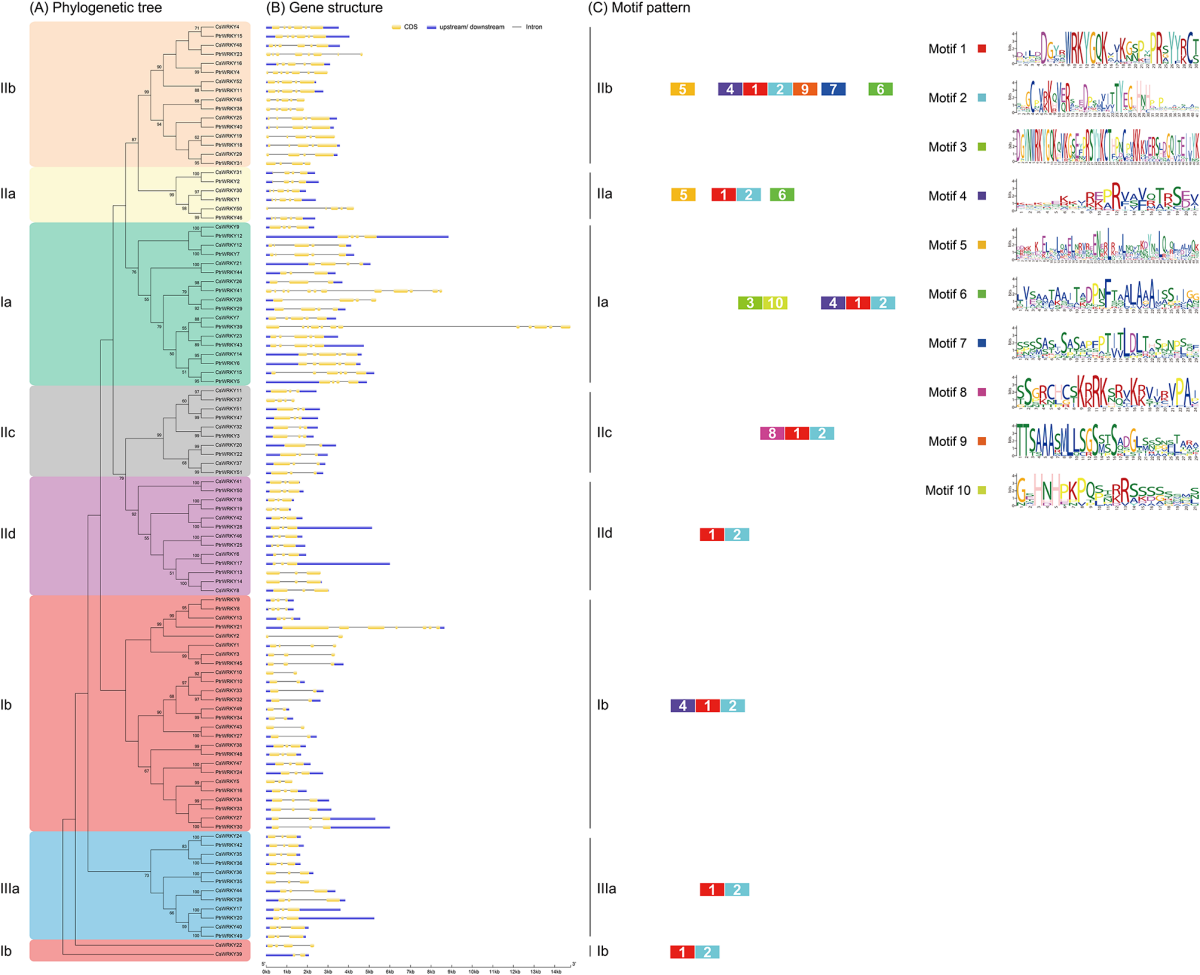
Gene structure and conserved motif analysis of *WRKY* genes in *Citrus sinensis* and *Poncirus trifoliata*. **(A)** Phylogenetic relationship of WRKY proteins from *C*. *sinensis* and *P*. *trifoliata*. **(B)** Exon/intron structures of *CsWRKYs* and *PtrWRKYs*. The exons and introns were represented by yellow boxes and black lines, respectively. The dark blue boxes indicated the upstream and/or downstream untranslated region. **(C)** The distribution of 10 conserved motifs in WRKY proteins, identified by MEME program, was shown by different colored blocks. The numbers 1–10 in the center of the blocks represent different motifs. The sequences of these conserved motifs were listed in Supplementary Table S4

The similarity and diversity of gene structures play prominent role in the evolution of gene family, and the positions of introns/exons are commonly well-conserved in orthologous genes. To investigate the structural diversity of the *CsWRKY* and *PtrWRKY* genes, we analyzed the distribution of introns/exons by comparing genomic and CDS sequences. The Group I genes have 2–6 exons in *C*. *sinensis* and 2–12 exons in *P*. *trifoliata*. All members from both citrus varieties of the Group IIa genes possess 4 or 5 exons. Variable numbers of exons were also found in Group IIb genes, 4–6 exons in *CsWRKY* genes, and 4–8 exons in *PtrWRKY* genes. In Group IIc members, 3 exons were found mostly in case of *CsWRKY* and *PtrWRKY* genes with Cs5g30250.4 (*CsWRKY20*) possessing 2 exons and Pt5g004720.1 (*PtrWRKY37*) possessing 4 exons, exceptionally. Overall, there was considerable diversity in the number of exons (2–12) and the length of exons in both citrus varieties. However, at the subfamily level, members exhibited similar gene structures in terms of exon number and length. For instance, all members of Group IId and Group IIIa contain 3 exons in both *C*. *sinensis* and *P*. *trifoliata* (Fig. 3A-B).

#### Conserved domain analysis

WRKY proteins usually possess additional conserved motifs that might be involved in activating the functions of WRKY proteins [43]. Therefore, other motifs may also serve unknown functional or structural roles along with the conserved residue of 60 amino acids of WRKY domain. The conserved motifs of CsWRKYs and PtrWRKYs were studied using the MEME program for structural diversity analysis and functional prediction. As a result, 10 conserved motifs were identified and the length of these motifs ranged from 21 to 50 amino acids (Supplementary Table S4). Amongst them, motif 1 and 3 were annotated as the WRKY domain. Out of the 8 remaining non-redundant motifs in CsWRKYs and PtrWRKYs, the function of the majority of motifs could not be predicted. Only two functional motifs of bZIP motif (motif 5) and plant-zinc cluster domain (motif 8) could be identified by MEME in both CsWRKY and PtrWRKY proteins. The 50 amino acid-long bZIP like motif denoted as motif 5, were distributed predominantly in Group IIa and Group IIb. The plant-zinc cluster domains of length 24 amino acids were explicitly presented in Group IIc. Except motif 1 and 2 being present in all the sequences, motif 5 and motif 6 are specifically and commonly shared by closely related Group IIa and Group IIb members. Similarly, motif 7 and motif 9 are unique to Group IIb members, as well as motif 3 and motif 10 only presented in Group Ia. The result suggesting the potential functions of these conserved motifs in their respective subgroups (Fig. 3C).

#### Chromosomal locations and gene duplication

**Fig. 4:**
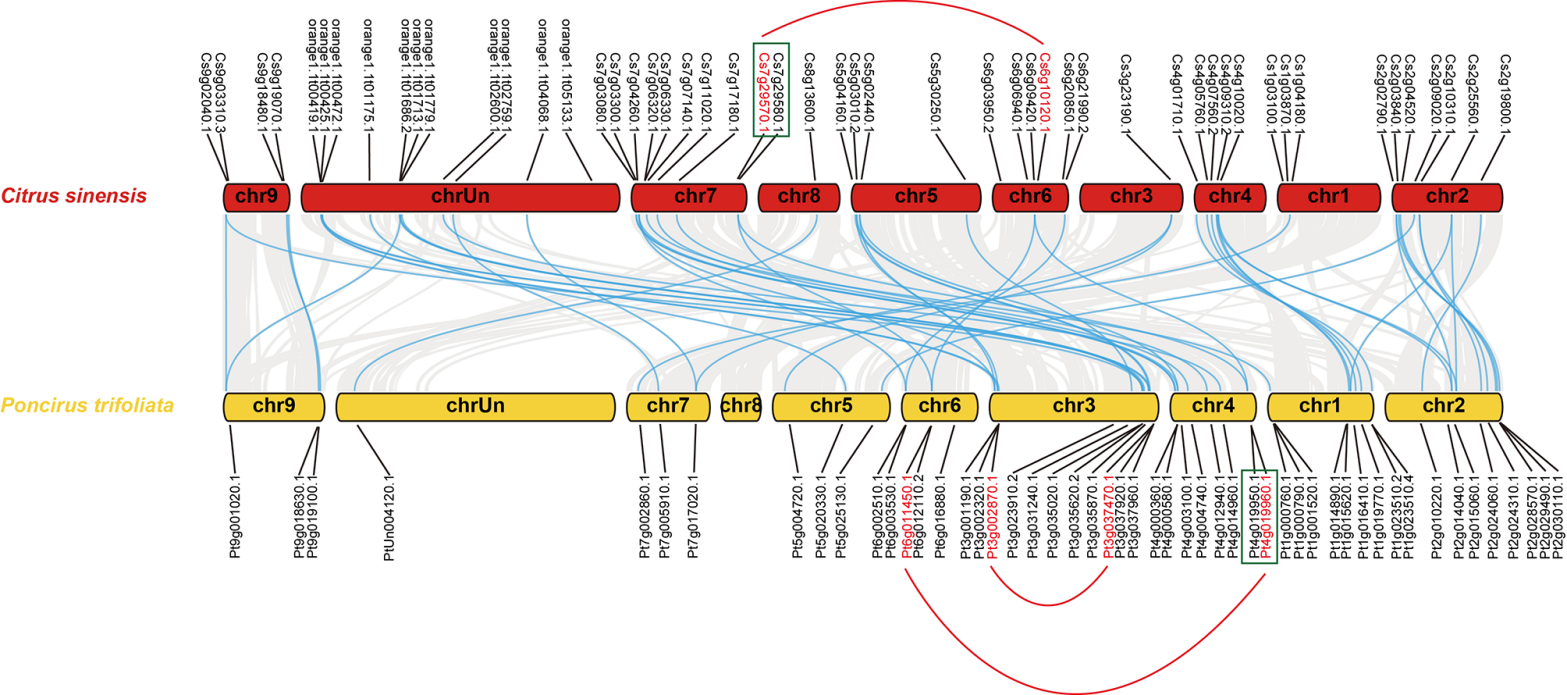
Chromosomal mapping and collinearity of *WRKY* genes among *Citrus sinensis* and *Poncirus trifoliata*. The horizontal columns represent chromosomes with the chromosome numbers placed in the middle and the gene ID shown outside the plot. The gray lines indicated the collinear blocks within these two species genomes, and the syntenic *WRKY* gene pairs were highlighted with the blue lines. Tandemly duplicated genes are represented by green blocks and the genes located on the duplicated segmental regions have been highlighted by red lines. The exact position of these *WRKY* genes was documented in Supplementary Table S2 and S3

In total, 52 and 51 members of *WRKY* genes were identified in the genome of *C*. *sinensis* and *P*. *trifoliata*, respectively. Out of 52, only 41 of *CsWRKY* genes could be mapped on the chromosome. The precise location of 11 genes could not be determined, which were orange1.1t00419.1 (*CsWRKY42*), orange1.1t00425.1 (*CsWRKY43*), orange1.1t00472.1 (*CsWRKY44*), orange1.1t01175.1 (*CsWRKY45*), orange1.1t01686.2 (*CsWRKY46*), orange1.1t01713.1 (*CsWRKY47*), orange1.1t01779.1 (*CsWRKY48*), orange1.1t02600.1 (*CsWRKY49*), orange1.1t02759.1 (*CsWRKY50*), orange1.1t04068.1 (*CsWRKY51*), and orange1.1t05133.1 (*CsWRKY52*). As shown in Fig. 4, distribution of *CsWRKY* genes was scattered throughout all nine chromosomes of *C*. *sinensis*. Most of the genes (10 genes, 19.2%) were located in chromosome 7, followed by chromosome 2 (7 genes, 13.5%), chromosome 6 (6 genes, 11.5%), chromosome 4 (5 genes, 9.6%), chromosome 5 (4 genes, 7.7%), chromosome 9 (4 genes, 7.7%) and chromosome 1 (3 genes, 5.8%). Chromosome 3 and 8 each has one gene.

In *P*. *trifoliata*, chromosome 3 had the largest number of *PtrWRKY* genes (11 genes, 21.6%), while chromosome 5, 7 and 9 had the smallest number (3 genes, 5.9%). Nine *PtrWRKY* genes were found on chromosomes 1 (17.6%). Eight *PtrWRKY* genes were distributed on both chromosomes 2 and 4 (15.7%) followed by five on chromosomes 6 (9.8%). Only one gene, PtUn004120.1 (*PtrWRKY51*), could not be located in any definite chromosome. Interestingly, no *PtrWRKY* was detected in chromosome 8 (Fig. 4).

It is well established that gene duplication plays a crucial role in the expansion of gene families in plant evolution. To further examine the evolution of *WRKY* genes in both varieties of citrus, genome duplication events were investigated for segmental and tandem duplications. Totally, only one pair of tandem duplication, Cs7g29570.1 (*CsWRKY35*) and Cs7g29580.1 (*CsWRKY36*) could be detected on chromosome 7 in *C*. *sinensis* (represented by green block in Fig. 4). In addition, two *CsWRKY* genes located on the duplicated segmental regions of chromosomes made up to two segmental duplication events (Cs6g10120.1/*CsWRKY24* and Cs7g29570.1/*CsWRKY35*) (represented by red line in Fig. 4). Similar result was found in the *P*. *trifoliata* genomes, the *PtrWRKY* genes, Pt4g019960.1 (*PtrWRKY36*) and Pt4g019950.1 (*PtrWRKY35*), were observed to be tandem duplicated on chromosome 4 (represented by green block in Fig. 4). Four *PtrWRKY* genes located on the duplicated segmental regions of chromosomes made up to two pairs of segmental duplications (Pt3g002870.1/*PtrWRKY20* and Pt3g037470.1/*PtrWRKY26*; Pt4g019960.1/*PtrWRKY36* and Pt6g011450.1/*PtrWRKY42* represented by red line in Fig. 4). In both *C*. *sinensis* and *P*. *trifoliata*, the tandem and segmental gene duplication events are not that significant, suggesting that these phenomena may play much insignificant role in the evolution of *CsWRKY* and *PtrWRKY* genes. Moreover, orthologous relationships of *WRKYs* between *C*. *sinensis* and *P*. *trifoliata* were analyzed, 48 orthologous gene pairs were identified (represented by blue line in Fig. 4). A total of seven *WRKY* genes could not found orthologous genes between *C*. *sinensis* and *P*. *trifoliata* (four genes from *C*. *sinensis* and three genes from *P*. *trifoliata*). It is worth noting that except one gene (Pt2g024060.1/*PtrWRKY13*) in *P*. *trifoliata*, the other six *WRKY* genes (Cs1g03100.1/*CsWRKY1*, Cs1g03870.1/*CsWRKY2*, Cs6g06940.1/*CsWRKY22*, Pt1g023510.4/*PtrWRKY9*, Pt3g023910.2/*PtrWRKY21*) were all belonged to Group Ib.

#### Expression profiles of WRKY genes under SA treatment

**Fig. 5:**
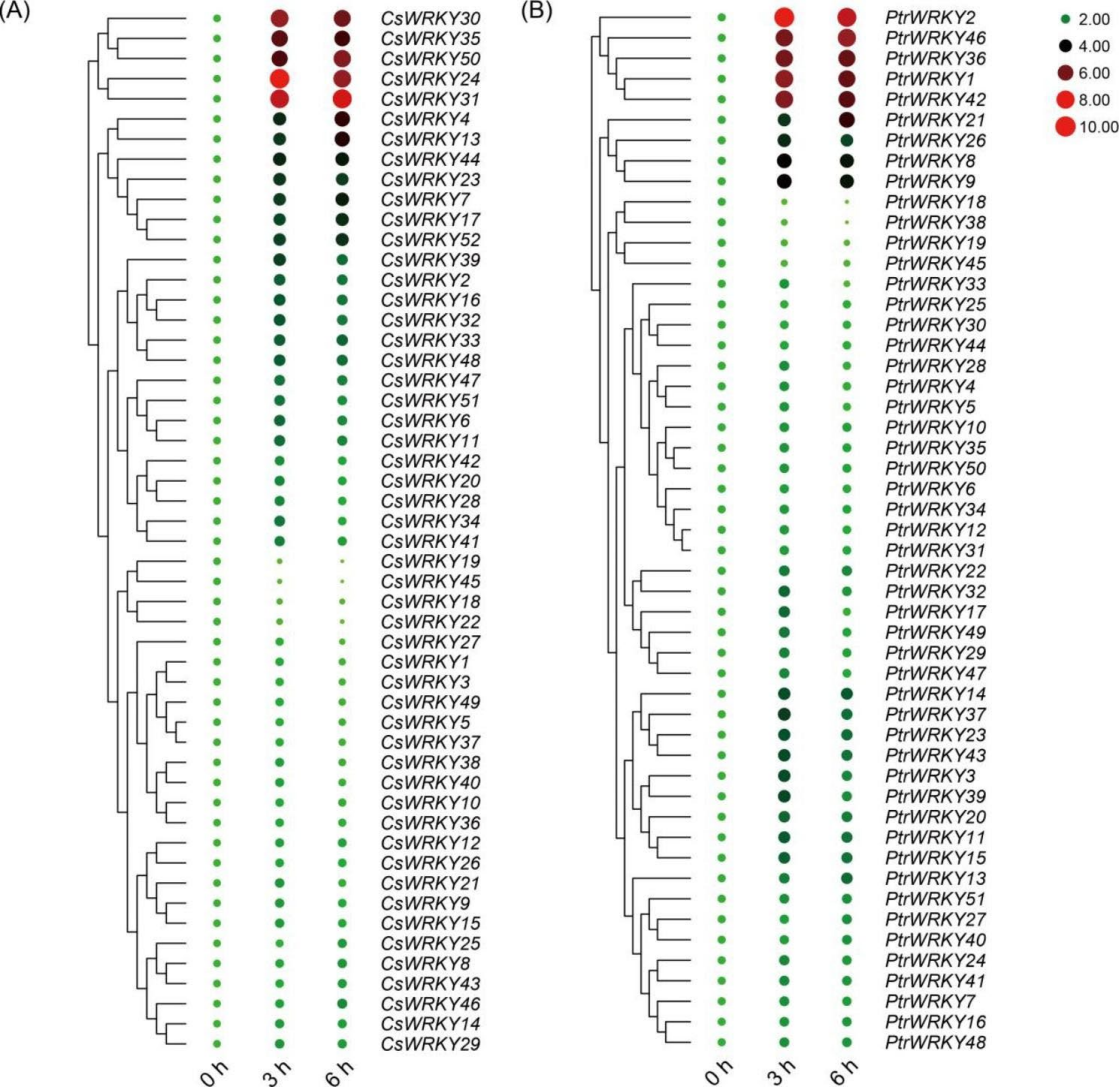
Expression profiles of *WRKY* genes of *Citrus sinensis* **(A)** and *Poncirus trifoliata * **(B)** under SA treatment. Expression analysis was carried out in leaves of *C*. *sinensis* and *P*. *trifoliata* at different time points (0 h, 3 and 6 h after irrigation of 2 mM SA). The qPCR results of *WRKY* genes were normalized by log_2_ transform. The heatmap constructed by TBtools software. Color scale erected vertically at the right side of the diagram. Higher expression levels are shown in red, bigger shape and lower expression levels are shown in green, smaller shape

To gain insights into the potential functions of the *WRKY* genes in plant disease resistance, we analyzed the expression patterns of all *CsWRKYs* and *PtrWRKYs* under SA treatment by qRT-PCR. Of the 52 *WRKY* genes in *C*. *sinensis*, 17 genes (*CsWRKY1*, *3*, *5*, *9*, *10*, *12*, *14*, *15*, *25*, *26*, *27*, *36*, *37*, *38*, *40*, *43*, and *49*) have no remarkable differences in transcript abundance, while the other 35 *CsWRKY* genes showed alteration of transcript levels in response to the SA treatment. The expression patterns of the genes varied among different members. Thirty-one *CsWRKY* genes (*CsWRKY2*, *4*, *6*, *7*, *8*, *11*, *13*, *16*, *17*, *20*, *21*, *23*, *24*, *28*, *29*, *30*, *31*, *32*, *33*, *34*, *35*, *39*, *41*, *42*, *44*, *46*, *47*, *48*, *50*, *51*, and *52*) showed increased expression levels during the SA treatment, while four *CsWRKY* genes (*CsWRKY18*, *19*, *22*, and *45*) were down-regulated by SA treatment (Fig. 5A, Supplementary Table S5).

The *WRKY* genes in *P*. *trifoliata* exhibited a similar expression pattern to that in *C*. *sinensis*. Most of the *PtrWRKY* genes (*PtrWRKY1*, *2*, *3*, *8*, *9*, *11*, *13*, *14*, *15*, *17*, *20*, *21*, *22*, *23*, *24*, *26*, *27*, *28*, *29*, *32*, *36*, *37*, *39*, *41*, *42*, *43*, *46*, *47*, and *49*) were significantly induced by SA treatment. Only *PtrWRKY18*, *19*, and *38* showed markedly decreased expression levels under SA treatment, and the remaining 10 *PtrWRKY* genes showed no conspicuous changes in their expression levels (Fig. 5B, Supplementary Table S5).

#### Expression profiles of WRKY genes under CLas-infection

**Fig. 6:**
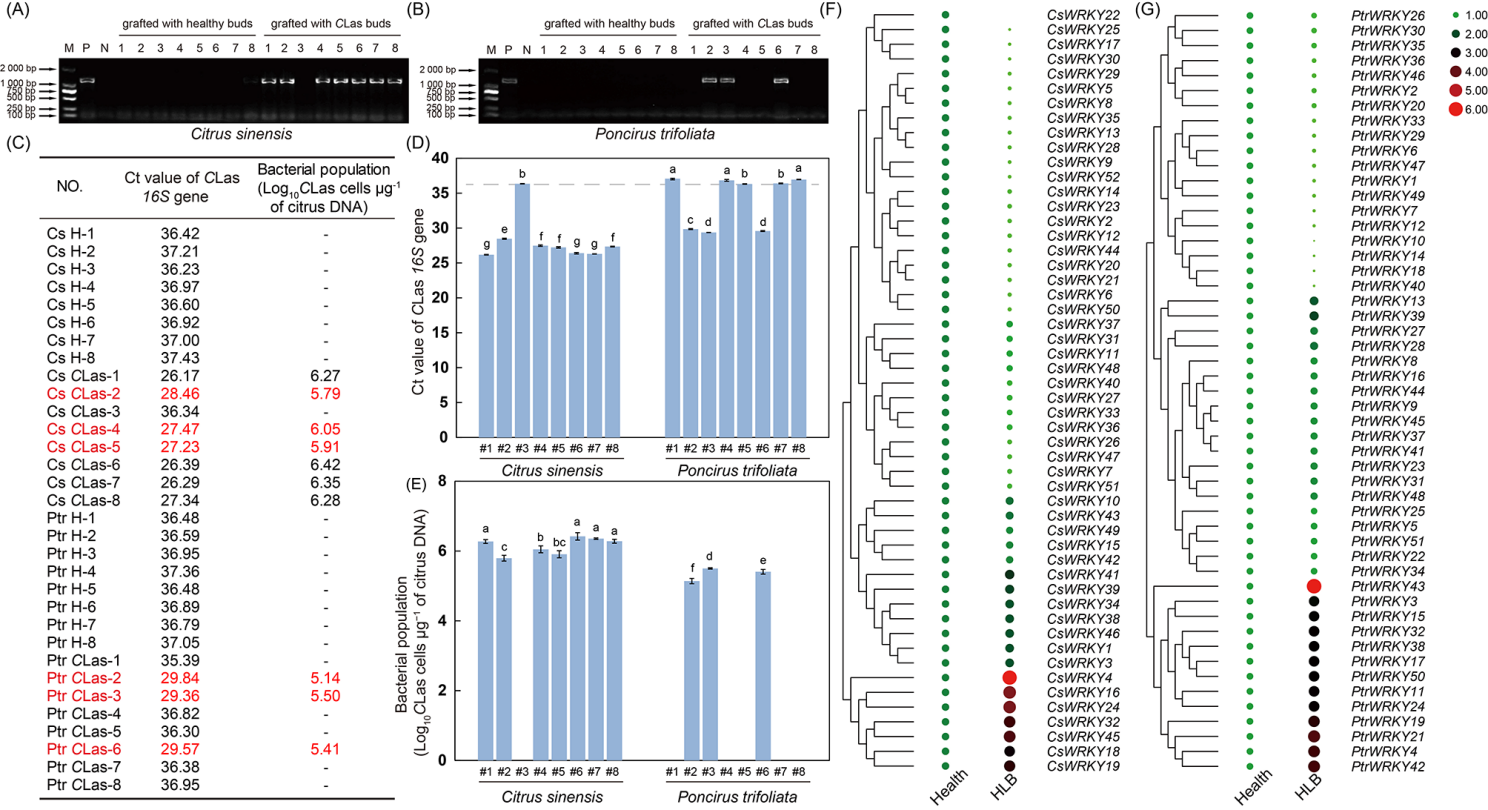
The detection of the *C* Las *16S* gene and the expression profiles of *WRKY* genes before and after *C* Las-inoculation. (A-B) The PCR determination results of the *C*Las pathogens at three months after grafting in *Citrus sinensis* seedlings **(A)** and *Poncirus trifoliata* seedlings **(B)**. Full-length gels are presented in Supplementary Figure S2. **(C-D)** The mean threshold cycle values (Ct) at three months after grafting. Different letters on top of the bars indicate significant differences between the highest Ct value of *C*Las *16 S* gene (#1 for the *P*. *trifoliata*) and the others based on a Tukey’s test (*p* < 0.05). **(E)** The bacterial populations (Log_10_*C*Las cells µg^− 1^ of citrus DNA) at three months after grafting. Different letters on the top of the column indicate significant differences between the highest bacterial population (#6 for the *C*. *sinensis*) and the others based on a Tukey’s test (*p* < 0.05). H, Healthy plant; *C*Las, *C*Las-inoculated plant. **(F-G)** Expression analysis was carried out in leaves of *C*. *sinensis* **(F)** and *P*. *trifoliata * **(G)** three months after inoculation. The qPCR results of *WRKY* genes were normalized by log_2_ transform. The heatmap constructed by TBtools software. Color scale erected vertically at the right side of the diagram, Higher expression levels are shown in red, bigger shape and lower expression levels are shown in green, smaller shape

To further determine the function of the *WRKY* genes in response to HLB disease, efforts were made to analyze expression patterns of all *WRKY* genes from *C*. *sinensis* and *P*. *trifoliata* in health and *C*Las-infected plants. After three months of *C*Las-inoculation via grafting, seven of the eight *C*. *sinensis* seedlings were identified as positive plants by PCR amplification, while only three of the *P*. *trifoliata* seedlings were detected with the presence of the *C*Las pathogen (Fig. 6A-B, Full-length gels are presented in Supplementary Figure S2). Further qPCR analysis showed that the *C*Las *16 S* Ct values of positive *C*. *sinensis* seedlings were significantly lower than those of positive *P*. *trifoliata* seedlings. Meanwhile, the bacterial populations of *C*Las of the grafted *C*. *sinensis* seedlings were also considerably higher than those of *P*. *trifoliata* seedlings (Fig. 6C-E). These results verified previous researches that *P*. *trifoliata* has a relative tolerance to HLB compared with *C*. *sinensis*. Materials from *C*Las-inoculated plants of *C*. *sinensis* (*C*Las-2, 4 and 5) and *P*. *trifoliata* (*C*Las-2, 3 and 6) with similar bacterial populations of *C*Las were selected for gene expression analysis. A considerable amount of *WRKY* genes (*CsWRKY2, 5, 6, 7, 8, 9, 12, 13, 14, 17, 20, 21, 22, 23, 25, 26, 27, 28, 29, 30, 33, 35, 36, 40, 44, 47, 50, 51*, and *52*) in *C*. *sinensis* displayed notably decreased expression levels in *C*Las-infected plants than that in healthy plants. Eight *CsWRKY* genes (*CsWRKY4*, *16*, *18*, *19*, *24*, *32*, *41*, and *45*) were remarkably induced in *C*Las-infected plants, while 15 *CsWRKY* genes (*CsWRKY1*, *3*, *10*, *11*, *15*, *31*, *34*, *37*, *38*, *39*, *42*, *43*, *46*, *48*, and *49*) showed no alteration of transcript levels between healthy and *C*Las-infected plants (Fig. 6F, Supplementary Table S6).

In *P*. *trifoliata*, 20 *PtrWRKY* genes (*PtrWRKY5*, *8*, *9*, *16*, *22*, *23*, *25*, *26*, *27*, *28*, *30*, *31*, *34*, *35*, *37*, *41*, *44, 45*, *48*, and *51*) showed no response to *C*Las-infection, 15 genes (*PtrWRKY3, 4*, *11*, *13*, *15*, *17*, *19*, *21*, *24*, *32*, *38*, *39*, *42*, *43*, and *50*) were up-regulated and 16 genes (*PtrWRKY1*, *2*, *6*, *7*, *10*, *12*, *14*, *18*, *20*, *29*, *33*, *36*, *40*, *46*, *47*, and *49*) were down-regulated after *C*Las-infection (Fig. 6G, Supplementary Table S6).

#### Differential WRKY genes expression analysis

**Fig. 7:**
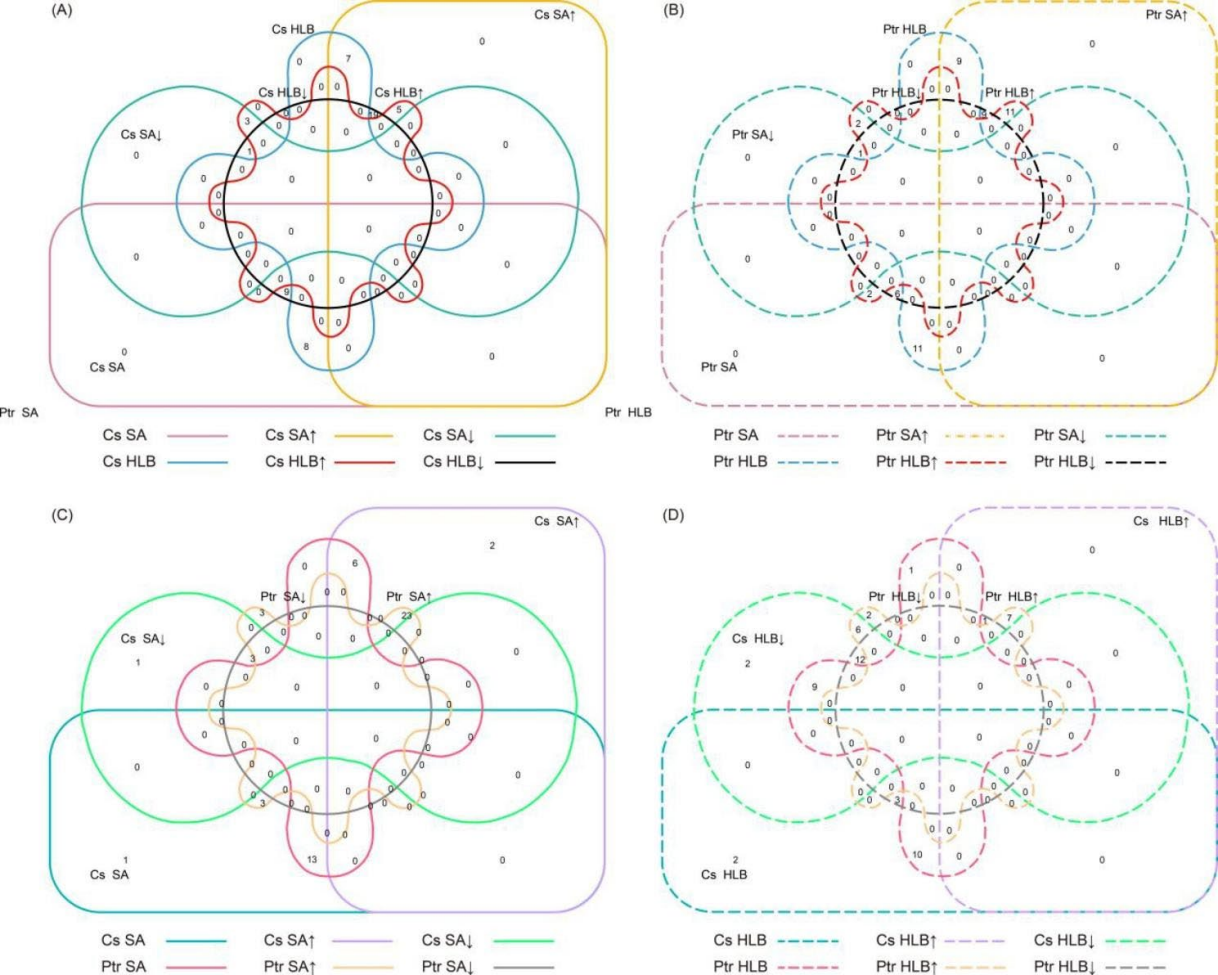
Venn diagram of *WRKY* genes of *Citrus sinensis* and *Poncirus trifoliata* under SA treatment and/or HLB infection. **(A)** Diagram of overlapping *CsWRKYs* under SA treatment and HLB infection. **(B)** Diagram of overlapping *PtrWRKYs* under SA treatment and HLB infection. **(C)** Diagram of overlapping *CsWRKYs* and *PtrWRKYs* under SA treatment. **(D)** Diagram of overlapping *CsWRKYs* and *PtrWRKYs* under HLB infection. Lines with different colors and traits represent different categories. Cs, *C*. *sinensis*; Ptr, *P*. *trifoliata*; SA↑, up-regulated by SA treatment; SA↓, down-regulated by SA treatment; SA, no significant change by SA treatment; HLB↑, up-regulated by HLB infection; HLB↓, down-regulated by HLB infection; HLB, no significant change by HLB infection

The similar expression patterns of *WRKY* gene under SA treatment and *C*Las-infection were likely related to the response of HLB in SA-dependent pathway. Firstly, a venn diagram was used to reveal the common expression changes among the two treatments in *C*. *sinensis* or *P*. *trifoliata*, separately. Six categories of SA↑ (up-regulated by SA treatment), SA↓ (down-regulated by SA treatment), SA (no significant change by SA treatment), HLB↑ (up-regulated by *C*Las-infection), HLB↓ (down-regulated by *C*Las-infection), and HLB (no significant change by *C*Las-infection) were performed. As shown in Fig. 7A, five up-regulated *CsWRKY* genes overlapped in the SA↑ and HLB↑ common comparisons, and one down-regulated *CsWRKY* gene was detected between SA↓ and HLB↓ common comparisons. Similarly, eleven common *PtrWRKY* genes were found to be up-regulated in the SA↑ and HLB↑ comparisons and one *PtrWRKY* gene was down-regulated in both SA↓ and HLB↓ categories (Fig. 7B). Further alignment of these *WRKY* genes revealed that three pairs of *WRKY* genes (*CsWRKY4*/*PtrWRKY15*, *CsWRKY24*/*PtrWRKY42*, and *CsWRKY32*/*PtrWRKY3*) from SA↑ and HLB↑ comparisons were orthologous genes, suggesting that these genes may be involved in the SA-dependent resistance to HLB.

The opposite expression patterns of *WRKY* genes across varieties were probably related to differences in tolerance to HLB. Furthermore, we identified the *WRKY* genes with inverse expression patterns between two varieties under SA treatment or *C*Las-infection, respectively. No common genes represented opposite expression pattern was observed between *C*. *sinensis* and *P*. *trifoliata* under SA treatment (Fig. 7C). However, six pairs of orthologous *WRKY* genes (*CsWRKY6*/*PtrWRKY17*, *CsWRKY7*/*PtrWRKY39*, *CsWRKY23*/*PtrWRKY43*, *CsWRKY33*/*PtrWRKY32*, *CsWRKY47*/*PtrWRKY24*, and *CsWRKY52*/*PtrWRKY11*) overlapped in the Cs HLB↓ and Ptr HLB↑ comparisons, and one pair of orthologous *WRKY* gene (*CsWRKY19*/*PtrWRKY18*) was detected between Cs HLB↑ and Ptr HLB↓ comparisons (Fig. 7D). These results suggest that the opposite expression *WRKY* genes under *C*Las-infection may contribute to the variation of HLB resistance between different citrus varieties.

#### Prediction of putative stress-responsive WRKYs and analysis of their promoters

**Fig. 8:**
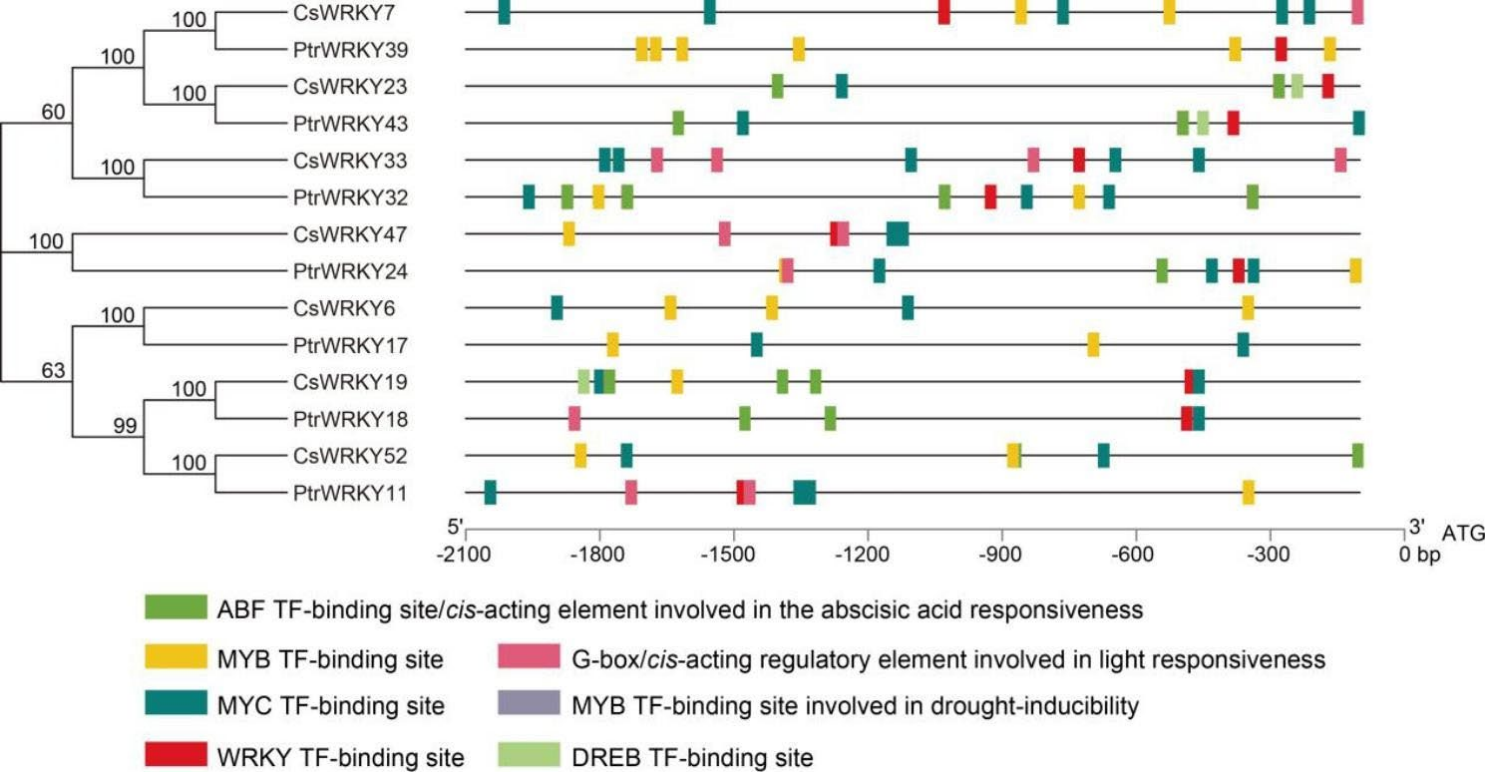
*Cis*-elements analysis in the promoters of candidate *WRKY* genes. Stress-responsive *cis*-elements were identified in the 2.0 kb upstream promoter region of *WRKY* genes candidates. Different colored rectangles represent different elements. Detailed information of sequence and position of these elements was described in Supplementary Tables S7

The interaction between TF and the stress-inducible *cis*-elements present in the promoter modulates the expression of gene regulatory networks response to the respective physical, environmental and biological stress [44]. As the orthologous *WRKY* genes exposed opposite expression patterns between *C*. *sinensis* and *P*. *trifoliata* under *C*Las-infection, we speculate that these alterations may be related to their upstream regulatory factors. To verify this assumption, we investigated the *cis*-elements in the promoter of seven pairs of orthologous *WRKY* genes with opposite expression patterns (six pairs from Cs HLB↓/Ptr HLB↑ comparisons, one pair from Cs HLB↑/Ptr HLB↓ comparisons). Subsequently, the − 2.0 kb promoter regions of these candidate *WRKY* genes were analyzed to identify stress-responsive *cis*-elements (Fig. 8, Supplementary Table S7). Among them, the promoter elements of three pairs of orthologous *WRKY* genes (*CsWRKY23*/*PtrWRKY43*, *CsWRKY47*/*PtrWRKY24*, and *CsWRKY6*/*PtrWRKY17*) were not significantly different in both type or quantity. There was little difference among two pairs of orthologous *WRKY* genes. For instance, *CsWRKY19* has a MYB-TF-binding site in its promoter region, while *PtrWRKY18* hasn’t. Similarly, an ABF-TF-binding site was captured in the promoter of *CsWRKY52*, but its’ orthologous gene, *PtrWRKY11*, contains no such *cis*-element in the promoter. Noteworthily, there exist five MYC-TF-binding sites in the promoter of *CsWRKY7*, while no existence of MYC-TF-binding site could be detected in the promoter of the orthologous gene *PtrWRKY39*. Conversely, *PtrWRKY32* contains four ABF-TF-binding sites in the promoter region whereas no ABF-TF-binding site was detected in the promoter of *CsWRKY33*.

#### Subcellular localization and transcriptional activation activity

**Fig. 9:**
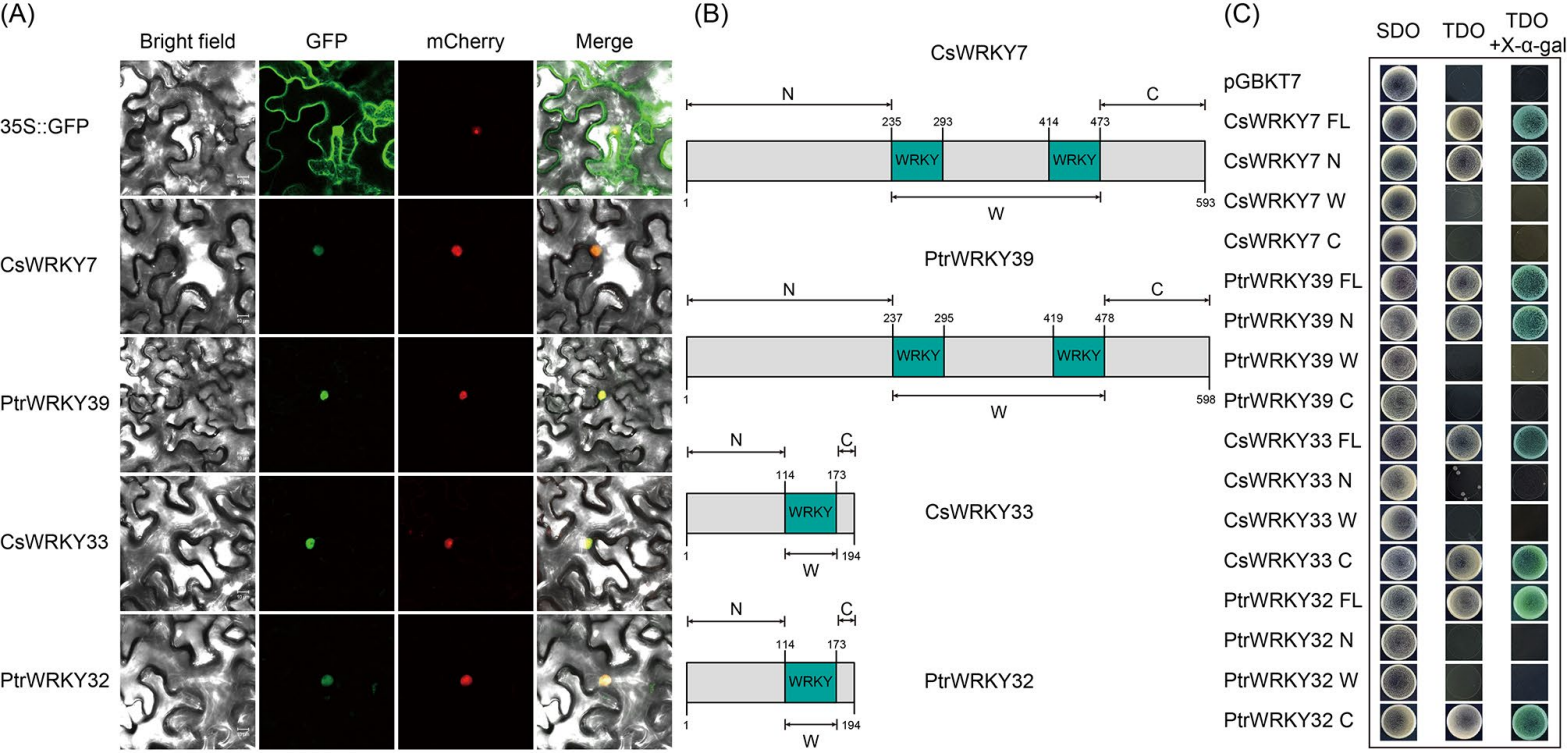
Subcellular localization and transcriptional activation assay of selected WRKY proteins. **(A)** The 35 S::GFP and 35 S::GFP-WRKY plasmids were transiently infiltrated into *Nicotiana benthamiana* leaves by *Agrobacterium tumefaciens*-mediated transformation and observed under a confocal laser scanning microscope. **(B)** Schematic structure of the four WRKY proteins. **(C)** The empty vector (pGBKT7) constructs and fusion constructs (FL or truncated fragments) were transformed into yeast Y2HGold strain and incubated on SD/-Trp (SDO), SD/-Trp/-His/-Ade (TDO) and TDO added with X-α-gal

Typical TFs usually have two basic characteristics: located in the nucleus and have transcriptional activation activity. For deeper study, we cloned two pairs of orthologous *WRKY* genes (*CsWRKY7*/*PtrWRKY39*, and *CsWRKY33*/*PtrWRKY32*) with opposite expression patterns under *C*Las-infection and significant differences in either type or quantity of *cis*-elements in promoter regions. Meanwhile, their subcellular localizations were analyzed by fusing the full length cDNAs with green fluorescent protein (GFP) under the drive of the CaMV 35 S promoter. Transient expression experiment was performed by *Agrobacterium*-mediated infiltration of *Nicotiana benthamiana* leaves. Co-transformation of a nucleus marker gene fused to mCherry was conducted to confirm the localization of nucleus. Control imaging of the epidermis of tobacco showed that the green fluorescence filled throughout the entire cell, including the membrane, the cytoplasm and the cell nucleus. The green fluorescence signals of four WRKY proteins, CsWRKY7, PtrWRKY39, CsWRKY33 and PtrWRKY32, were exclusively distributed in the nucleus (Fig. 9A).

To determine the transcriptional activation activity of these four nuclear localized WRKY proteins in gene expression regulation, the GAL4-responsive reporter system in yeast was carried out. Four tested WRKY proteins were truncated into N-terminal (N), W-terminal (containing the conserved WRKY domain, W) and C-terminal (C) according to the amino acid structure (Fig. 9B). The full length (FL) and three truncated fragments (N, W, and C) of *WRKYs* were fused to pGBKT7 to generate effectors, respectively. All the fusion plasmids were independently transformed into the Y2HGold yeast strain and exhibited visible white colonies on the SD medium lacking tryptophan (SD/-Trp, SDO). All yeast transformants containing FL fragments of CsWRKY7, PtrWRKY39, CsWRKY33, and PtrWRKY32 survived on the SD/-Trp/-Ade/-His medium (TDO) and exhibited blue pigment on the SD/-Trp/-Ade/-His medium added with X-α-Gal (TDO + X-α-Gal). Among all truncated fragments, only yeast cells transformed with the effectors containing CsWRKY7-N, PtrWRKY39-N, CsWRKY33-C and PtrWRKY32-C grew and displayed GAL4 activity on the medium supplemented with X-α-gal (Fig. 9C). These results demonstrate that the four WRKY proteins have transcriptional activation activity, among which CsWRKY7 and PtrWRKY39 exhibit strong transcriptional activation ability at its N-terminal respectively, and the C-terminal is necessary for the transactivation of CsWRKY33 and PtrWRKY32.

## Discussion

In this study, gene architectures, protein motifs, gene duplications and syntenic relationships of the WRKY family were globally analyzed in two *Citrus* species. Furthermore, the expression pattern and functional analysis of the *WRKY* genes were further investigated.

### Genome-wide exploration and evolutionary analysis of ***WRKY*** genes among two ***Citrus*** species

A BLASTp search and HMMER analysis revealed a total of 52 WRKY proteins in *C*. *sinensis* genome and 51 WRKY proteins in *P*. *trifoliata* genome. Previous research excavated 50 non-redundant *WRKY* genes in sweet orange by using “WRKY & Citrus” as keywords to search within ten databases [45]. Detailed comparison exhibited that all of the 50 *CsWRKYs* were included in the *WRKY* genes identified in our study, and two more *CsWRKYs* (orange1.1t01175.1 and orange1.1t02759.1) were discovered in our results compared with the previous article, suggesting that our approach of BLASTp search after constructing the Hidden Markov Model could identify potential *WRKY* genes more comprehensively and completely. In both *C*. *sinensis* and *P*. *trifoliata*, most genes were discovered to be closely related to each other due to similarities in their intron-exon structures, indicating their close phylogenetic relationship. This remarkable resemblance in intron-exon structure also supports the concept of a common ancestry [46, 47]. It was also observed in both species that the intron number of the overwhelming majority of *WRKY* genes was higher in Group IIb than in other Groups. Similar findings in *Arabidopsis*, sesame, sorghum, and sugar beet were reported [20, 48–50]. A recent study demonstrated a higher frequency of intron loss than the frequency of intron gain after segmental duplication in rice [51]. These results suggesting that Group Ia subfamily might have the original genes, from which other clusters were derived. Furthermore, the lowest number of introns were present in Group IIc, indicating that *WRKY* genes from Group IIc in *Citrus* might be in a posterior position in evolution. Other species i.e. *Chenopodium quinoa* and *Isatis indigotica* also exhibited the same feature [52, 53].

The conserved motif analysis revealed interesting facts regarding the gene evolution. The motif 4 of Group I proteins, occurring just before the motif 1 containing WRKY domain, were also found in Group IIb proteins (Fig. 3), indicating that the Group IIb genes have been originated from the loss of the N-terminal WRKY domain of Group I genes. Additionally, the phylogenetic closeness and the conserved motifs of Group Ib, Group IId and Group IIIa indicate their evolution from a common origin. Notably, collinearity analysis exhibited that seven *WRKY* genes from *C*. *sinensis* and *P*. *trifoliata* had no orthologous genes between each other, among which six *WRKY* genes belonged to Group Ib (Fig. 4). Furthermore, five WRKY proteins containing a mutated WRKY domain of WRKYGKK with Q amino acid mutation to K amino acid all belonged to Group Ib (Supplementary Figure S1). Combined with the analysis of collinearity, we speculated that *WRKY* genes from Group Ib in *Citrus* might be in a relatively anterior position in evolution. Whereas, most members of other subgroups may have not evolved from the subgroup. Thus, our data support the theory of evolution of WRKY genes that the Group I is the oldest group, and Group II along with Group III have been evolved from Group I. In addition, our study further investigated that WRKY members of Group II and Group III may have been evolved from Group Ia in *Citrus*.

According to the research on the evolution of *WRKY* family between *Arabidopsis* and rice, some of the rice *WRKY* genes in Group III are evolutionarily more active than those in *Arabidopsis*, suggesting that evolution is more active in the Group III and these members of the group may have more functions in the monocotyledonous plants [42]. This variation in the distribution of WRKY genes among monocotyledons and dicotyledons suggests that members of the Group III have been evolved independently after the dissection of the monocotyledons and dicotyledons. It’s worth mentioning that neither *C. sinensis* nor *P. trifoliata* has any members classified into Group IIIb. On the basis of the number of amino acid residues between the inner amino acids of the C2H zinc finger motifs, Group III was divided into two subgroups as Groups IIIa (n ≤ 23) and IIIb (n ≥ 24) respectively. Therefore, the deletion of Group IIIb *WRKY* genes in the genomes of two *Citrus* species might be due to the shortening of amino acids of these genes during evolution, which showed diversity in the length of WRKY domains.

Distribution of genes suggests unlike gene structure diversity of *WRKYs* in different species. Thus, the uneven distribution of genes is evidence of genetic variations resulting as an evolutionary process of these two species after divergence from each other [54]. The recent gene duplication events including segmental duplication and tandem duplication were thought to be key driving forces in the expansion and evolution of gene families, as well as the raw materials for new biological functions. Because of their considerable contribution to various physiological processes, the WRKY family in angiosperms is likely to expand rapidly during evolution. Recent gene duplication events have been reported to be more extensive in the expansion of WRKY genes in many crops, such as rice and *Arabidopsis* [42]. Nonetheless, in some cases, recent duplications seemed to play no substantial role in *WRKY* gene expansion [55, 56]. In our case also, we detected few tandem and segmental gene duplications in two *Citrus* species, *C*. *sinensis* and *P*. *trifoliata*, but not that conspicuous as compared to rice and *Arabidopsis*. Notably, we observed that all pairs of tandem-duplicated genes in both *C*. *sinensis* and *P*. *trifoliata* genome belonged to Group IIIa, which is consistent with a previous study on *Arabidopsis* [42]. Our study revealed that tandem duplication events of Group III *WRKY* gene family were considered as main contributors to the rapid expansion of *Citrus* species *WRKY* gene family.

### The expression and promoter analysis of ***WRKY*** genes provide important clues for their function under ***C***Las-infection

Previous studies have shown that *WRKYs* involved in pathogenic mechanism in plants. SA is a vital hormone in the immunological responses to pathogens. Several *WRKY* genes have been reported to be induced by SA and play a crucial role in plant disease resistance. CaWRKY6 transcriptionally activated *CaWRKY40* and positively regulated *Ralstonia solanacearum* resistance in pepper [24]. VqWRKY31 was confirmed to fight against powdery mildew pathogen attack in grapevine [57]. The mechanism of their resistance to pathogen infection was all related to SA signaling. In our current study, expression pattern analysis of WRKY family genes from *C*. *sinensis* and *P*. *trifoliata* showed that most of the genes displayed different up- and down-regulation patterns in response to SA treatment and *C*Las-infection. Several genes showed a significant induction multiple. For example, the expression levels of *CsWRKY24* reached 259.17 and 92.20 times at 3 and 6 h after SA treatment, respectively (Fig. 5, Supplementary Table S5). *CsWRKY4* and *PtrWRKY43* separately exhibited an enhanced expression level of 17.99-fold and 48.56-fold compared to healthy plants (Fig. 6, Supplementary Table S6), suggesting that some *WRKY* genes could be stimulated by SA treatment or *C*Las-infection. Further investigation revealed a high percentage of overlap between *WRKY* genes which expressed dramatically after *C*Las-infection and SA treatment. Previous studies have shown that homologous genes of these *WRKY* genes function in the SA-mediated plant disease resistance. For instance, AtWRKY6 (a homologous gene of *CsWRKY4*/*PtrWRKY15*) positively influenced the pathogen defense-associated *PR1* promoter activity, most likely involving NPR1 function due to the elevated transcription levels of *NPR1* in *Arabidopsis thaliana* [58]. *AtWRKY70* (a homologous gene of *CsWRKY24*/*PtrWRKY42*) have been identified as a node of convergence for integrating SA- and JA-mediated signaling events during plant response to bacterial pathogens. Gain or loss function of WRKY70 led to opposite effects on JA-mediated resistance to *A. brassicicola* and the SA-mediated resistance to *E. cichoracearum*, suggesting that it had a vital role in balancing SA-dependent and JA-dependent defense pathways [59]. Given that orthologous genes among different plants are generally considered to retain similar functions and to share other key properties, we hypothesize that these homologous *WRKY* genes in *Citrus* may likewise play a critical role in the SA-induced resistance pathway to HLB. Notably, *AtWRKY11*, the homologous gene of last *Citrus WRKY* orthologous gene pairs, has been discovered to be a negative regulator of the basal resistance to *Pseudomonas syringae* pv *tomato* (*Pst*). Loss function of AtWRKY11 enhanced resistance to both avirulent and virulent *Pst* strains [60]. Another study showed that two *Arabidopsis* TFs, AtWRKY11 and AtWRKY70, were identified as essential regulators in Induced Systemic Resistance (ISR) triggered by *Bacillus cereus* AR156. AR156 treatment dramatically induced the transcription of *AtWRKY70*, but inhibited that of *AtWRKY11* in *Arabidopsis* leaves. AtWRKY11 regulated AR156 triggering ISR by activating JA signaling pathway, while AtWRKY70 regulated ISR by activating SA signaling pathway [61]. These researches indicate that there are multiple regulatory pathways in the mechanisms of *WRKY* genes in disease resistance signaling pathway.

To analyze the difference of *WRKY* gene in SA signaling and HLB response between disease tolerant and susceptible varieties, we screened *WRKY* genes with opposite expression patterns after two treatments in sweet orange and trifoliate orange, resulting in the identification of seven pairs of homologous *WRKY* genes (Fig. 7C-D), suggesting that these genes may play a pivotal role in the differences in susceptibility of citrus varieties to HLB disease. The *cis*-elements present in the promoter regions are important molecular switches that control a wide range of gene regulatory networks [62]. Our analysis of these seven pairs of homologous *WRKY* genes revealed significant differences in the promoter *cis*-elements of two homologous *WRKY* gene pairs (*CsWRKY7*/*PtrWRKY39*, *CsWRKY33*/*PtrWRKY32*). Previous studies showed that *AtWRKY33* (a homologous gene of *CsWRKY7*/*PtrWRKY39*) participates in the SA signal pathway and plays a negative regulatory role in resisting *Botrytis cinerea* concomitant with increased expression of SA-regulated *PR-1* gene, and reduced expression of JA-regulated genes such as *PDF1.2* in *wrky33* mutant plants [63]. However, the specific upstream regulatory factors of *WRKY33* are still not clear. WRKY45 (a homologous gene of *CsWRKY33*/*PtrWRKY32* in rice) is an important TF in the SA signaling pathway, which mediates chemically induced resistance to a variety of pathogens [64]. One research revealed that the TF Ideal Plant Architecture 1 (IPA1) was phosphorylated at Ser163 within its DNA binding domain along with the alteration of DNA binding specificity after the infection of *Magnaporthe oryzae*. Phosphorylated IPA1 binds to the promoter of *WRKY45* and activates its expression, thereby enhancing disease resistance in rice [65]. These findings imply that the disease resistance conferred by WRKY45 might be closely related to its promoter activity and upstream regulators. In conclusion, the above findings suggest that the opposite expression patterns of two pairs of homologous *WRKY* genes (*CsWRKY7*/*PtrWRKY39*, *CsWRKY33*/*PtrWRKY32*) may also be related to their upstream regulatory factors between disease-tolerant and disease-susceptible citrus varieties.

### The candidate ***WRKYs*** are typical TFs

A close relationship between the function of TFs and their localization has been demonstrated by numerous studies [66]. In the present study, the two screened pairs of homologous *WRKY* genes, *CsWRKY7*, *PtrWRKY39*, *CsWRKY33* and *PtrWRKY32* were all localized in the nucleus (Fig. 9A). Transcriptional activation analysis showed that all the four WRKY proteins exhibited transcriptional activity in yeast cells (Fig. 9C), demonstrating that these WRKY proteins are typical TFs with transcriptional activation regulatory ability and function in the nucleus, thus implying that their functions may be related to the direct regulatory effects on downstream target genes. However, more in-depth studies are needed to elucidate the upstream regulators and potential downstream target genes in SA signaling pathway and *C*Las-infection. Our results provide comprehensive information for the function of *WRKY* genes in *Citrus* and take important significance in the identification of HLB resistance genes as well as the investigation of molecular mechanism and regulatory network in response to HLB and regulated by SA signaling pathway.

## Conclusion

A total of 52 and 51 *WRKY* genes were identified and analyzed in two *Citrus* species: *C. sinensis* and *P. trifoliata*, respectively. qRT-PCR and promoter analysis showed that the expressions of 2 pairs of candidate orthologous *WRKY* genes were opposite between two *Citrus* species, and these genes might be involved in the HLB response and SA signaling pathway. Experiments indicated that these *WRKY* genes (*CsWRKY7*/*PtrWRKY39*, *CsWRKY33*/*PtrWRKY32*) are classic WRKY TFs with transcriptional regulation function localized in the nucleus.

## Electronic supplementary material

Below is the link to the electronic supplementary material.

**Additional file 1: Table S1.** List of primer sequences used in this study

**Additional file 2: Table S2.** Proposed nomenclature and important features of WRKYs from *Citrus sinensis*

**Additional file 3: Table S3.** Proposed nomenclature and important features of WRKYs from *Poncirus trifoliata*

**Additional file 4: Figure S1.** Multiple sequence alignment of WRKY domains in CsWRKYs and PtrWRKYs

**Additional file 5: Table S4.** Conserved motifs in CsWRKY and PtrWRKY proteins.

**Additional file 6: Table S5.** qRT-PCR values of *WRKY* genes under SA treatment

**Additional file 7: Figure S2.** The uncropped gel of Fig. 6A-B.

**Additional file 8: Table S6.** qRT-PCR values of *WRKY* genes under HLB infection

**Additional file 9: Table S7.** List of stress-responsive *cis*-acting elements present in 2 kb upstream region of *WRKY* genes

## Data Availability

The sequence information of *Citrus* and *Arabidopsis* WRKY family genes were collected from Citrus Pan-genome to Breeding Database (http://citrus.hzau.edu.cn/index.php) and The Arabidopsis Information Resource (https://www.arabidopsis.org/) respectively. All data used during the current study are included in this published article and its supplementary information files or available from the corresponding author on reasonable request.

## List of Abbreviations

aa: Sequence, amino acid
ACP: Asian Citrus Psyllid
*C*Las: *Candidatus* Liberibacter asiaticus
CTAB: Cetyltrimethylammonium Bromide
FL: Full Length
GFP: Green Fluorescent Protein
HLB: Huanglongbing
ISR: Induced Systemic Resistance
ORF: Open Reading Frame
qPCR: Quantitative Polymerase Chain Reaction
qRT-PCR: Quantitative real-time Polymerase Chain Reaction
SA: Salicylic Acid
SAR: Systemic Acquired Resistance
TF: Transcription Factor
