# Supplementary material for: Genome-wide identification and comparative expression profiling of the WRKY transcription factor family in two Citrus species with different Candidatus Liberibacter asiaticus susceptibility
Source: BMC Plant Biol. 2023 Mar 24;23:159. doi: 10.1186/s12870-023-04156-4 (PMC10037894; doi:10.1186/s12870-023-04156-4)
Supplement: Supplementary file 1 — Additional file 1: Table S1. List of primer sequences used in this study [file 12870_2023_4156_MOESM1_ESM.docx]

**Additional file 1: Table S1. List of primer sequences used in this study**

| **Purpose** | **Primer names** | **Primer sequences (5’-3’)** | |  |
| --- | --- | --- | --- | --- |
|  |  | **Forward primer** | **Reverse primer** | |
| *C*Las detection | *C*Las *16S* | \| GCGCGTATGCAATACGAGCGGCA \| \| --- \| | \| GCCTCGCGACTTCGCAACCCAT \| \| --- \| | |
| *C*Las quantification | q*C*Las 16S | TGAGTGCTAGCTGTTGGGTG | CTGCGCGTTGCATCGAATTA | |
|  | qCt 18S | AATTGTTGGTCTTCAACGAGGAA | AAAGGGCAGGGACGTAGTCAA | |
| Gene expression | *CsWRKY1* qRT | CAGCAGGTGCAGGTGTAGG | GGGCAGAGTTTTCGGTGGAG | |
|  | *CsWRKY2* qRT | CGAACAAAATCGGAGCTGGA | AGGTCCAGCCATTTTGGGG | |
|  | *CsWRKY3* qRT | CTTCACAAGCCATGTTGAACC | GCAATGAAAGAAGTTGGGCC | |
|  | *CsWRKY4* qRT | GACAAAGGATGGGGTCTCACA | ACTATTGTCGGAGGCCGGAA | |
|  | *CsWRKY5* qRT | CGCCTTCTTCATCCGAAGTG | TGATTGCAGGGCTTGGTCAT | |
|  | *CsWRKY6* qRT | TGCGGATGAATTCACGTGGT | GATTTCTGTGGCGGCGAAAC | |
|  | *CsWRKY7* qRT | AGAGAACCGGGTCCGGTGTG | TGGAGATGGCAGAACGTTGG | |
|  | *CsWRKY8* qRT | ACCCCTTGCCCACCTGGCAG | TGGTGGTGGAGCGTAATAGC | |
|  | *CsWRKY9* qRT | GGATTGGATGAAGTTGCTTCCG | GATCCGATGGCAGCAAGTGA | |
|  | *CsWRKY10* qRT | TGTTCATCTACAGGGAAATCGGT | TCAGACCCATGAAAAGCTCCA | |
|  | *CsWRKY11* qRT | GGTAGACCAAGAACAGGGCA | GACGATGAACAGGGGTAGGG | |
|  | *CsWRKY12* qRT | GAGGCAACTCACTGAGCCAC | ACCGCTATACTTCCCGAACC | |
|  | *CsWRKY13* qRT | ACCACAGGACTCACCTGAGA | CCTTCGAAGTGGGTGCTAGT | |
|  | *CsWRKY14* qRT | GCTCAAGAGAACCACCCAGT | CATTAAACCCAGCTCTTGCCGC | |
|  | *CsWRKY15* qRT | CTGAGGTTCGAGTGGGTGAC | AACGACGACGGAGAAAGACC | |
|  | *CsWRKY16* qRT | TAAGTTGGCAGTTCTCCGGG | TATCATCTCACGGCCTGCTG | |
|  | *CsWRKY17* qRT | TAGTGGTGTACCGCCAGAGT | GTCCTTCAAGGCCACTCTCA | |
|  | *CsWRKY18* qRT | TCGAGTCTGCAACAAGATGTGA | GAATCGGAAAGCTTAGGCCG | |
|  | *CsWRKY19* qRT | TGCTGAGCATCCGGTACTAAC | TTGCGTTTGAAGGGTCTGGT | |
|  | *CsWRKY20* qRT | TCGGGTCATGCAAGAGTGAG | TCCAAAGATGGGTTGCCCAG | |
|  | *CsWRKY21* qRT | GTGCCTTCAAGACCTACTCGT | TTTGGCGGAGCACAATTCAC | |
|  | *CsWRKY22* qRT | AAGACGACGAGCTTGTGAGG | AGTTGGCCCCGAATATACAGC | |
|  | *CsWRKY23* qRT | CCAACTCTGTTTCAGTGGAGGA | ACCAGATTGAGGAGCAGCAT | |
|  | *CsWRKY24* qRT | TGACTCTGATGAAGCGTCCC | GTTCTGTCCATGACTCCGCA | |
|  | *CsWRKY25* qRT | GAAATTAGCAGCCGACGGGA | TAGATTTCTTGGCGGGGTCA | |
|  | *CsWRKY26* qRT | GATGGAGATGGGGACGACAAG | CTGTAACTGTAGCCAACGCC | |
|  | *CsWRKY27* qRT | CAGTCGAGCTGGAAGCTAGG | GGATTTGTCAAACCGCCACC | |
|  | *CsWRKY28* qRT | CACTCCTTCACGACCGACAA | CGGCGAGTAGCTGAGAGAAG | |
|  | *CsWRKY29* qRT | AGCTGTCGTTATCAAAAATGGCG | CATCCTTCTTAGGCTGCGCT | |
|  | *CsWRKY30* qRT | CAATTTGCGGCAGCAGTACA | GGCCTTTTGCATGACTCTTCG | |
|  | *CsWRKY31* qRT | TGAGAATCTGAGGCATCTGGTG | GGAGGAACTTGTGCTGTGACT | |
|  | *CsWRKY32* qRT | CTCTACGGCGAGTTACGTCC | TCCGGTGATAGCAGAGGACA | |
|  | *CsWRKY33* qRT | AACATATTTTCCAACAGCGGCG | CCGGATTAACTTCCATCCCCG | |
|  | *CsWRKY34* qRT | TGGACGACACTCCCAAGAAG | GCGCCAGCTTCGTTAGATGA | |
|  | *CsWRKY35* qRT | AAGATGGGTTGGTTTCGGCT | CCGGCGATAGTCATCGGAAT | |
|  | *CsWRKY36* qRT | AGCTTTGTTTTATCCGCCCG | GGGGTTCTCCCAACGAAGTG | |
|  | *CsWRKY37* qRT | TTCAACAGCAACACATGGGC | GAACCCCCATCCAAGCTCAA | |
|  | *CsWRKY38* qRT | ACTTGCTCAATGTCCAGAACCA | TGAGTTGGGAGTAGCTGGGT | |
|  | *CsWRKY39* qRT | GGTTCACAGTCACACCCACC | CTAATTGCGTCTGCTAGCTGGT | |
|  | *CsWRKY40* qRT | TTCAGCTCAACTTGCGGACT | TCAGGGGACTCCCGGTTAAA | |
|  | *CsWRKY41* qRT | TCCGTTTGTTATTGGGGAGCA | ATCGATGGGCATGTGATGGC | |
|  | *CsWRKY42* qRT | GTGGTCAGAGGCTGCAACAG | CTTCGAAAGGATCGGCCAAG | |
|  | *CsWRKY43* qRT | TTCGTCGATGCAACCAGCTA | TCTTGTTGATCACGACCTCCG | |
|  | *CsWRKY44* qRT | GAAAGAGCTAGCTAAGCGGC | GGGCTAGTAGCAGTGTTGGG | |
|  | *CsWRKY45* qRT | CAAGTGAAATTGGCCGCGAT | TGCTGATAGGGGACTTTCGT | |
|  | *CsWRKY46* qRT | TCACCCATAAGGTGTCCTCA | CGTACTGGGCAGTTCTCCAA | |
|  | *CsWRKY47* qRT | TTCTCAGCTTCGAGGCGTAT | ACGGAACCTGATTCTGTGCC | |
|  | *CsWRKY48* qRT | CTGGCACATGGGAGCCCTCA | GTCATCGGATACCGAAGGCT | |
|  | *CsWRKY49* qRT | ATGGGAATTGATCCCCGTGC | ATGTCAACCTGGCTTCTCGT | |
|  | *CsWRKY50* qRT | CGTTAACAGCGTTGTGCGAGAG | CCCATTGTTAGCGCAACATTG | |
|  | *CsWRKY51* qRT | ATTATGCGAGGCCGATCCAG | ACCAGTCACAGTCAGCGAAG | |
|  | *CsWRKY52* qRT | GTGAACGCGGAGAATCAACG | TCTATAAACCGCCTCGGTGC | |
|  | *PtrWRKY1* qRT | CAATTTGCGGCAGCAGTACA | GGCCTTTTGCATGACTCTTCG | |
|  | *PtrWRKY2* qRT | TGAGAATCTGAGGCATCTGGTG | GGAGGAACTTGTGCTGTGACT | |
|  | *PtrWRKY3* qRT | CTCTACGGCGAGTTACGTCC | TCCGGTGATAGCAGAGGACA | |
|  | *PtrWRKY4* qRT | TAAGTTGGCAGTTCTCCGGG | TATCATCTCACGGCCTGCTG | |
|  | *PtrWRKY5* qRT | CTGAGGTTCGAGTGGGTGAC | AACGACGACGGAGAAAGACC | |
|  | *PtrWRKY6* qRT | GCTCAAGAGAACCACCCAGT | CATTAAACCCAGCTCTTGCCGC | |
|  | *PtrWRKY7* qRT | GAGGCAACTCACTGAGCCAC | ACCGCTATACTTCCCGAACC | |
|  | *PtrWRKY8* qRT | ACCACAGGACTCACCTGAGA | CCTTCGAAGTGGGTGCTAGT | |
|  | *PtrWRKY9* qRT | ACCACAGGACTCACCTGAGA | CCTTCGAAGTGGGTGCTAGT | |
|  | *PtrWRKY10* qRT | TGTTCATCTACAGGGAAATCGGT | TCAGACCCATGAAAAGCTCCA | |
|  | *PtrWRKY11* qRT | GTGAACGCGGAGAATCAACG | TCTATAAACCGCCTCGGTGC | |
|  | *PtrWRKY12* qRT | GGATTGGATGAAGTTGCTTCCG | GATCCGATGGCAGCAAGTGA | |
|  | *PtrWRKY13* qRT | GGGGATTTGACTGATATTATTC | GAAGCCGCTGTTGCTGCTTC | |
|  | *PtrWRKY14* qRT | ACCCCTTGCCCACCTGGCAG | TGGTGGTGGAGCGTAATAGC | |
|  | *PtrWRKY15* qRT | GACAAAGGATGGGGTCTCACA | ACTATTGTCGGAGGCCGGAA | |
|  | *PtrWRKY16* qRT | CGCCTTCTTCATCCGAAGTG | TGATTGCAGGGCTTGGTCAT | |
|  | *PtrWRKY17* qRT | TGCGGATGAATTCACGTGGT | GATTTCTGTGGCGGCGAAAC | |
|  | *PtrWRKY18* qRT | TGCTGAGCATCCGGTACTAAC | TTGCGTTTGAAGGGTCTGGT | |
|  | *PtrWRKY19* qRT | TCGAGTCTGCAACAAGATGTGA | GAATCGGAAAGCTTAGGCCG | |
|  | *PtrWRKY20* qRT | TAGTGGTGTACCGCCAGAGT | GTCCTTCAAGGCCACTCTCA | |
|  | *PtrWRKY21* qRT | GCATTGGGAAGACGACACTGC | CTCCAAATCAAGGCTTGTCC | |
|  | *PtrWRKY22* qRT | TCGGGTCATGCAAGAGTGAG | TCCAAAGATGGGTTGCCCAG | |
|  | *PtrWRKY23* qRT | CTGGCACATGGGAGCCCTCA | GTCATCGGATACCGAAGGCT | |
|  | *PtrWRKY24* qRT | TTCTCAGCTTCGAGGCGTAT | ACGGAACCTGATTCTGTGCC | |
|  | *PtrWRKY25* qRT | TCACCCATAAGGTGTCCTCA | CGTACTGGGCAGTTCTCCAA | |
|  | *PtrWRKY26* qRT | GAAAGAGCTAGCTAAGCGGC | GGGCTAGTAGCAGTGTTGGG | |
|  | *PtrWRKY27* qRT | TTCGTCGATGCAACCAGCTA | TCTTGTTGATCACGACCTCCG | |
|  | *PtrWRKY28* qRT | GTGGTCAGAGGCTGCAACAG | CTTCGAAAGGATCGGCCAAG | |
|  | *PtrWRKY29* qRT | CACTCCTTCACGACCGACAA | CGGCGAGTAGCTGAGAGAAG | |
|  | *PtrWRKY30* qRT | CAGTCGAGCTGGAAGCTAGG | GGATTTGTCAAACCGCCACC | |
|  | *PtrWRKY31* qRT | AGCTGTCGTTATCAAAAATGGCG | CATCCTTCTTAGGCTGCGCT | |
|  | *PtrWRKY32* qRT | AACATATTTTCCAACAGCGGCG | CCGGATTAACTTCCATCCCCG | |
|  | *PtrWRKY33* qRT | TGGACGACACTCCCAAGAAG | GCGCCAGCTTCGTTAGATGA | |
|  | *PtrWRKY34* qRT | ATGGGAATTGATCCCCGTGC | ATGTCAACCTGGCTTCTCGT | |
|  | *PtrWRKY35* qRT | AGCTTTGTTTTATCCGCCCG | GGGGTTCTCCCAACGAAGTG | |
|  | *PtrWRKY36* qRT | AAGATGGGTTGGTTTCGGCT | CCGGCGATAGTCATCGGAAT | |
|  | *PtrWRKY37* qRT | GGTAGACCAAGAACAGGGCA | GACGATGAACAGGGGTAGGG | |
|  | *PtrWRKY38* qRT | CAAGTGAAATTGGCCGCGAT | TGCTGATAGGGGACTTTCGT | |
|  | *PtrWRKY39* qRT | AGAGAACCGGGTCCGGTGTG | TGGAGATGGCAGAACGTTGG | |
|  | *PtrWRKY40* qRT | GAAATTAGCAGCCGACGGGA | TAGATTTCTTGGCGGGGTCA | |
|  | *PtrWRKY41* qRT | GATGGAGATGGGGACGACAAG | CTGTAACTGTAGCCAACGCC | |
|  | *PtrWRKY42* qRT | TGACTCTGATGAAGCGTCCC | GTTCTGTCCATGACTCCGCA | |
|  | *PtrWRKY43* qRT | CCAACTCTGTTTCAGTGGAGGA | ACCAGATTGAGGAGCAGCAT | |
|  | *PtrWRKY44* qRT | GTGCCTTCAAGACCTACTCGT | TTTGGCGGAGCACAATTCAC | |
|  | *PtrWRKY45* qRT | CTTCACAAGCCATGTTGAACC | GCAATGAAAGAAGTTGGGCC | |
|  | *PtrWRKY46* qRT | CGTTAACAGCGTTGTGCGAGAG | CCCATTGTTAGCGCAACATTG | |
|  | *PtrWRKY47* qRT | ATTATGCGAGGCCGATCCAG | ACCAGTCACAGTCAGCGAAG | |
|  | *PtrWRKY48* qRT | ACTTGCTCAATGTCCAGAACCA | TGAGTTGGGAGTAGCTGGGT | |
|  | *PtrWRKY49* qRT | TTCAGCTCAACTTGCGGACT | TCAGGGGACTCCCGGTTAAA | |
|  | *PtrWRKY50* qRT | TCCGTTTGTTATTGGGGAGCA | ATCGATGGGCATGTGATGGC | |
|  | *PtrWRKY51* qRT | TTCAACAGCAACACATGGGC | GAACCCCCATCCAAGCTCAA | |
| Internal control | *Actin* | CATCCCTCAGCACCTTCC | CCAACCTTAGCACTTCTCC | |
| Subcellular Localization | pBI121-EGFP-CsWRKY7 | TCTAGAATGGCCTCTTCTTCTGGTAACTTAG | CCCGGGAAATAGCAATGACTCGAAG | |
|  | pBI121-EGFP-PtrWRKY39 | TCTAGAATGGCCTCTTCTTCTGGTAACTTAG | CCCGGGAAATAGCAATGACTCGAAG | |
|  | pBI121-EGFP-CsWRKY33 | TCTAGAATGGAGAATTACCAAACATAT | CCCGGGATAAGAAGTGTAGATTTGCATC | |
|  | pBI121-EGFP-PtrWRKY32 | TCTAGAATGGAGAATTACCAAACATAT | CCCGGGATAAGAAGTGTAGATTTGCATC | |
| Transcriptional Activation Activity | pGBKT7-CsWRKY7-FL | GAATTCATGGCCTCTTCTTCTGGTAAC | GGATTCAAATAGCAATGACTCGAAGA | |
|  | pGBKT7-CsWRKY7-N | GAATTCATGGCCTCTTCTTCTGGTAAC | GGATTCTTTTTTACTCTCTCTAATGGGC | |
|  | pGBKT7-CsWRKY7-W | GAATTCTCTGATGATGGATATAATTGG | GGATTCAACATCATGGTTGTGCTTTC | |
|  | pGBKT7-CsWRKY7-C | GAATTCCCGGCAGCTCGCGGCAGCGG | GGATTCAAATAGCAATGACTCGAAGA | |
|  | pGBKT7-PtrWRKY39-FL | GAATTCATGGCCTCTTCTTCTGGTAAC | GGATTCAAATAGCAATGACTCGAAGA | |
|  | pGBKT7-PtrWRKY39-N | GAATTCATGGCCTCTTCTTCTGGTAAC | GGATTCTTTTTTACTCTCTCTAATGGGC | |
|  | pGBKT7-PtrWRKY39-W | GAATTCTCTGATGATGGATATAATTGG | GGATTCAACATCATGGTTGTGCTTTC | |
|  | pGBKT7-PtrWRKY39-C | GAATTCCCGGCAGCTCGCGGCAGCGG | GGATTCAAATAGCAATGACTCGAAGA | |
|  | pGBKT7-CsWRKY33-FL | GAATTCATGGAGAATTACCAAACATAT | GGATTCATAAGAAGTGTAGATTTGCATC | |
|  | pGBKT7-CsWRKY33-N | GAATTCATGGAGAATTACCAAACATAT | GGATTCTATATCAACTTGGCTTCGTG | |
|  | pGBKT7-CsWRKY33-W | GAATTCCTTGATGATGGCTATAGATG | GGATTCGATGGGATGTGAATGCATTC | |
|  | pGBKT7-CsWRKY33-C | GAATTCGAGAGATCAACTGATAACTTC | GGATTCATAAGAAGTGTAGATTTGCATC | |
|  | pGBKT7-PtrWRKY32-FL | GAATTCATGGAGAATTACCAAACATAT | GGATTCATAAGAAGTGTAGATTTGCATC | |
|  | pGBKT7-PtrWRKY32-N | GAATTCATGGAGAATTACCAAACATAT | GGATTCTATATCGACTTGGCTTCTTG | |
|  | pGBKT7-PtrWRKY32-W | GAATTCCTTGATGATGGCTATAGATG | GGATTCGATGGGATGTGAATGCATTC | |
|  | pGBKT7-PtrWRKY32-C | GAATTCGAGAAATCAACCGATAACTTC | GGATTCATAAGAAGTGTAGATTTGCATC | |
