# Supplementary material for: Genome-wide identification and comparative expression profiling of the WRKY transcription factor family in two Citrus species with different Candidatus Liberibacter asiaticus susceptibility
Source: BMC Plant Biol. 2023 Mar 24;23:159. doi: 10.1186/s12870-023-04156-4 (PMC10037894; doi:10.1186/s12870-023-04156-4)
Supplement: Supplementary file 2 — Additional file 2: Table S2. Proposed nomenclature and important features of WRKYs from Citrus sinensis [file 12870_2023_4156_MOESM2_ESM.docx]

**Additional file 2: Table S2. Proposed nomenclature and important features of WRKYs from *Citrus sinensis***

| **Gene name** | **Gene ID** | **Chromosome NO.** | **Strand (+ or -)** | **Start position** | **End position** | **Exon number** | **Intron number** | **Open readeing frame** | **Amino acid number** | **Relative molecular weight (kD)** | **Isoelectric point** | **Group** |
| --- | --- | --- | --- | --- | --- | --- | --- | --- | --- | --- | --- | --- |
| CsWRKY1 | Cs1g03100.1 | 1 | - | 2270797 | 2274197 | 4 | 3 | 726 | 242 | 27.08 | 7.05 | Ib |
| CsWRKY2 | Cs1g03870.1 | 1 | - | 3087282 | 3091014 | 2 | 1 | 348 | 116 | 13.35 | 9.15 | Ib |
| CsWRKY3 | Cs1g04180.1 | 1 | + | 3485909 | 3489254 | 3 | 2 | 693 | 231 | 25.81 | 9.22 | Ib |
| CsWRKY4 | Cs2g02790.1 | 2 | + | 1166737 | 1170265 | 6 | 5 | 1830 | 610 | 66.06 | 6.03 | IIb |
| CsWRKY5 | Cs2g03840.1 | 2 | - | 1809564 | 1810844 | 3 | 2 | 948 | 316 | 34.90 | 6.56 | Ib |
| CsWRKY6 | Cs2g04520.1 | 2 | - | 2278266 | 2280205 | 3 | 2 | 810 | 270 | 30.57 | 5.26 | IId |
| CsWRKY7 | Cs2g09020.1 | 2 | - | 6146166 | 6149568 | 5 | 4 | 1779 | 593 | 64.65 | 7.08 | Ia |
| CsWRKY8 | Cs2g10310.1 | 2 | - | 7627245 | 7630275 | 3 | 2 | 1518 | 506 | 54.38 | 5.60 | IId |
| CsWRKY9 | Cs2g19800.1 | 2 | - | 16597423 | 16599756 | 4 | 3 | 1467 | 489 | 53.31 | 6.27 | Ia |
| CsWRKY10 | Cs2g25560.1 | 2 | - | 24763856 | 24765366 | 2 | 1 | 552 | 184 | 20.88 | 8.80 | Ib |
| CsWRKY11 | Cs3g23190.1 | 3 | + | 25538725 | 25541177 | 3 | 2 | 1077 | 359 | 38.69 | 9.30 | IIc |
| CsWRKY12 | Cs4g01710.1 | 4 | + | 546928 | 550996 | 5 | 4 | 1458 | 486 | 53.28 | 6.18 | Ia |
| CsWRKY13 | Cs4g05760.1 | 4 | - | 3532847 | 3534502 | 3 | 2 | 480 | 160 | 18.30 | 5.29 | Ib |
| CsWRKY14 | Cs4g07560.2 | 4 | + | 4806432 | 4811078 | 5 | 4 | 2172 | 724 | 78.03 | 5.72 | Ia |
| CsWRKY15 | Cs4g09310.2 | 4 | - | 6195542 | 6200714 | 6 | 5 | 1692 | 564 | 60.90 | 6.58 | Ia |
| CsWRKY16 | Cs4g10020.1 | 4 | + | 6670189 | 6673283 | 5 | 4 | 1440 | 480 | 52.29 | 8.90 | IIb |
| CsWRKY17 | Cs5g02440.1 | 5 | + | 1080665 | 1084277 | 3 | 2 | 1011 | 337 | 37.64 | 5.20 | IIIa |
| CsWRKY18 | Cs5g03010.2 | 5 | - | 1488895 | 1490249 | 3 | 2 | 924 | 308 | 34.27 | 5.93 | IId |
| CsWRKY19 | Cs5g04160.1 | 5 | - | 2321902 | 2325229 | 5 | 4 | 1809 | 603 | 65.19 | 6.02 | IIb |
| CsWRKY20 | Cs5g30250.4 | 5 | - | 32172106 | 32175238 | 2 | 1 | 1176 | 392 | 44.22 | 9.62 | IIc |
| CsWRKY21 | Cs6g03950.2 | 6 | - | 4519015 | 4524087 | 4 | 3 | 1407 | 469 | 51.88 | 8.67 | Ia |
| CsWRKY22 | Cs6g06940.1 | 6 | + | 8833626 | 8835959 | 4 | 3 | 1047 | 349 | 39.28 | 6.04 | Ib |
| CsWRKY23 | Cs6g09420.1 | 6 | - | 11178822 | 11182318 | 5 | 4 | 1698 | 566 | 62.36 | 6.29 | Ia |
| CsWRKY24 | Cs6g10120.1 | 6 | - | 11786019 | 11787703 | 3 | 2 | 987 | 329 | 36.79 | 5.91 | IIIa |
| CsWRKY25 | Cs6g20850.1 | 6 | + | 20194206 | 20197649 | 5 | 4 | 1767 | 589 | 63.58 | 6.16 | IIb |
| CsWRKY26 | Cs6g21990.2 | 6 | + | 20895588 | 20899292 | 3 | 2 | 1593 | 531 | 58.71 | 7.97 | Ia |
| CsWRKY27 | Cs7g03080.1 | 7 | - | 1298173 | 1303121 | 3 | 2 | 876 | 292 | 32.05 | 6.19 | Ib |
| CsWRKY28 | Cs7g03300.1 | 7 | + | 1410331 | 1415689 | 4 | 3 | 1578 | 526 | 56.81 | 6.96 | Ia |
| CsWRKY29 | Cs7g04260.1 | 7 | - | 2126526 | 2129982 | 4 | 3 | 1704 | 568 | 62.79 | 6.80 | IIb |
| CsWRKY30 | Cs7g06320.1 | 7 | + | 3696558 | 3698483 | 5 | 4 | 963 | 321 | 35.77 | 6.80 | IIa |
| CsWRKY31 | Cs7g06330.1 | 7 | + | 3705792 | 3708168 | 4 | 3 | 777 | 259 | 28.81 | 8.94 | IIa |
| CsWRKY32 | Cs7g07140.1 | 7 | + | 4260845 | 4263354 | 3 | 2 | 1026 | 342 | 36.74 | 9.54 | IIc |
| CsWRKY33 | Cs7g11020.1 | 7 | - | 7242159 | 7244955 | 2 | 1 | 585 | 195 | 21.80 | 9.25 | Ib |
| CsWRKY34 | Cs7g17180.1 | 7 | - | 12654326 | 12657389 | 3 | 2 | 1029 | 343 | 38.27 | 6.60 | Ib |
| CsWRKY35 | Cs7g29570.1 | 7 | + | 29778597 | 29780253 | 3 | 2 | 930 | 310 | 35.00 | 6.01 | IIIa |
| CsWRKY36 | Cs7g29580.1 | 7 | - | 29782722 | 29785025 | 3 | 2 | 1071 | 357 | 39.28 | 6.20 | IIIa |
| CsWRKY37 | Cs8g13600.1 | 8 | - | 16355947 | 16358822 | 3 | 2 | 1080 | 360 | 40.54 | 9.83 | IIc |
| CsWRKY38 | Cs9g02040.1 | 9 | - | 671464 | 673389 | 3 | 2 | 1116 | 372 | 40.91 | 6.00 | Ib |
| CsWRKY39 | Cs9g03310.3 | 9 | - | 1610778 | 1612866 | 2 | 1 | 384 | 128 | 14.54 | 9.07 | Ib |
| CsWRKY40 | Cs9g18480.1 | 9 | + | 17646412 | 17648474 | 3 | 2 | 1149 | 383 | 42.24 | 5.55 | IIIa |
| CsWRKY41 | Cs9g19070.1 | 9 | - | 18136219 | 18137855 | 3 | 2 | 1212 | 404 | 44.53 | 5.06 | IId |
| CsWRKY42 | orange1.1t00419.1 | UN | + | 5566849 | 5568616 | 3 | 2 | 1041 | 347 | 37.78 | 6.32 | IId |
| CsWRKY43 | orange1.1t00425.1 | UN | + | 5588061 | 5589926 | 2 | 1 | 567 | 189 | 21.58 | 9.30 | Ib |
| CsWRKY44 | orange1.1t00472.1 | UN | - | 5888815 | 5892186 | 3 | 2 | 1095 | 365 | 41.11 | 5.23 | IIIa |
| CsWRKY45 | orange1.1t01175.1 | UN | - | 19266005 | 19267858 | 5 | 4 | 1185 | 395 | 44.09 | 5.83 | IIb |
| CsWRKY46 | orange1.1t01686.2 | UN | + | 27465432 | 27466953 | 3 | 2 | 825 | 275 | 31.08 | 5.12 | IId |
| CsWRKY47 | orange1.1t01713.1 | UN | + | 27672465 | 27674620 | 3 | 2 | 969 | 323 | 35.54 | 8.56 | Ib |
| CsWRKY48 | orange1.1t01779.1 | UN | - | 28119497 | 28123047 | 6 | 5 | 1506 | 502 | 54.41 | 8.58 | IIb |
| CsWRKY49 | orange1.1t02600.1 | UN | - | 39677835 | 39678955 | 2 | 1 | 489 | 163 | 18.27 | 9.56 | Ib |
| CsWRKY50 | orange1.1t02759.1 | UN | + | 42430326 | 42434578 | 5 | 4 | 960 | 320 | 35.43 | 8.35 | IIa |
| CsWRKY51 | orange1.1t04068.1 | UN | - | 63057402 | 63060016 | 3 | 2 | 1098 | 366 | 40.41 | 9.26 | IIc |
| CsWRKY52 | orange1.1t05133.1 | UN | + | 83231411 | 83233828 | 5 | 4 | 1800 | 600 | 65.17 | 6.28 | IIb |
