# Supplementary material for: Genome-wide identification and comparative expression profiling of the WRKY transcription factor family in two Citrus species with different Candidatus Liberibacter asiaticus susceptibility
Source: BMC Plant Biol. 2023 Mar 24;23:159. doi: 10.1186/s12870-023-04156-4 (PMC10037894; doi:10.1186/s12870-023-04156-4)
Supplement: Supplementary file 3 — Additional file 3: Table S3. Proposed nomenclature and important features of WRKYs from Poncirus trifoliata [file 12870_2023_4156_MOESM3_ESM.docx]

**Additional file 3: Table S3. Proposed nomenclature and important features of WRKYs from *Poncirus trifoliata***

| **Gene name** | **Gene ID** | **Chromosome NO.** | **Strand (+ or -)** | **Start position** | **End position** | **Exon number** | **Intron number** | **Open readeing frame** | **Amino acid number** | **Relative molecular weight (kD)** | **Isoelectric point** | **Group** |
| --- | --- | --- | --- | --- | --- | --- | --- | --- | --- | --- | --- | --- |
| PtrWRKY1 | Pt1g000760.1 | 1 | - | 1229961 | 1232362 | 5 | 4 | 966 | 322 | 35.88 | 7.68 | IIa |
| PtrWRKY2 | Pt1g000790.1 | 1 | - | 1220884 | 1223429 | 4 | 3 | 774 | 258 | 28.55 | 8.61 | IIa |
| PtrWRKY3 | Pt1g001520.1 | 1 | - | 571710 | 574031 | 3 | 2 | 1026 | 342 | 36.73 | 9.50 | IIc |
| PtrWRKY4 | Pt1g014890.1 | 1 | - | 20877720 | 20880689 | 7 | 6 | 1797 | 599 | 65.92 | 8.10 | IIb |
| PtrWRKY5 | Pt1g015620.2 | 1 | + | 21317494 | 21320934 | 4 | 3 | 1326 | 442 | 48.12 | 6.38 | Ia |
| PtrWRKY6 | Pt1g016410.2 | 1 | - | 22645244 | 22649826 | 5 | 4 | 2172 | 724 | 78.06 | 5.76 | Ia |
| PtrWRKY7 | Pt1g019770.2 | 1 | - | 26840263 | 26844539 | 5 | 4 | 1458 | 486 | 53.21 | 6.13 | Ia |
| PtrWRKY8 | Pt1g023510.2 | 1 | + | 24101090 | 24102425 | 4 | 3 | 486 | 162 | 18.37 | 5.32 | Ib |
| PtrWRKY9 | Pt1g023510.4 | 1 | + | 24101090 | 24102425 | 3 | 2 | 486 | 162 | 18.43 | 5.30 | Ib |
| PtrWRKY10 | Pt2g010220.1 | 2 | + | 9515015 | 9516888 | 2 | 1 | 552 | 184 | 20.97 | 9.33 | Ib |
| PtrWRKY11 | Pt2g014040.1 | 2 | - | 17158142 | 17160915 | 5 | 4 | 1800 | 600 | 65.24 | 6.45 | IIb |
| PtrWRKY12 | Pt2g015060.1 | 2 | + | 18339043 | 18347901 | 4 | 3 | 1467 | 489 | 53.13 | 6.33 | Ia |
| PtrWRKY13 | Pt2g024060.1 | 2 | + | 24777444 | 24780088 | 3 | 2 | 1488 | 496 | 53.45 | 5.84 | IId |
| PtrWRKY14 | Pt2g024310.1 | 2 | + | 26367685 | 26370377 | 3 | 2 | 1488 | 496 | 53.45 | 5.84 | IId |
| PtrWRKY15 | Pt2g028570.1 | 2 | - | 29506417 | 29510463 | 6 | 5 | 1833 | 611 | 66.06 | 6.03 | IIb |
| PtrWRKY16 | Pt2g029490.1 | 2 | + | 28948730 | 28950701 | 3 | 2 | 954 | 318 | 35.22 | 6.80 | Ib |
| PtrWRKY17 | Pt2g030110.1 | 2 | + | 28525796 | 28531795 | 3 | 2 | 792 | 264 | 29.78 | 5.20 | IId |
| PtrWRKY18 | Pt3g001190.1 | 3 | + | 1382406 | 1385981 | 5 | 4 | 1809 | 603 | 65.24 | 6.23 | IIb |
| PtrWRKY19 | Pt3g002320.1 | 3 | + | 2132258 | 2133461 | 3 | 2 | 894 | 298 | 33.36 | 5.66 | IId |
| PtrWRKY20 | Pt3g002870.1 | 3 | - | 2504872 | 2510132 | 3 | 2 | 1011 | 337 | 37.60 | 5.20 | IIIa |
| PtrWRKY21 | Pt3g023910.2 | 3 | - | 30162648 | 30171301 | 7 | 6 | 4170 | 1390 | 159.05 | 6.10 | Ib |
| PtrWRKY22 | Pt3g031240.1 | 3 | - | 36706790 | 36709777 | 3 | 2 | 1053 | 351 | 39.26 | 9.69 | IIc |
| PtrWRKY23 | Pt3g035020.1 | 3 | - | 39954356 | 39959034 | 8 | 7 | 1512 | 504 | 55.26 | 8.74 | IIb |
| PtrWRKY24 | Pt3g035620.2 | 3 | + | 39566743 | 39569505 | 3 | 2 | 975 | 325 | 35.76 | 8.77 | Ib |
| PtrWRKY25 | Pt3g035870.1 | 3 | + | 39397797 | 39399708 | 3 | 2 | 825 | 275 | 31.11 | 5.12 | IId |
| PtrWRKY26 | Pt3g037470.1 | 3 | + | 41089839 | 41093683 | 3 | 2 | 1095 | 365 | 41.05 | 5.23 | IIIa |
| PtrWRKY27 | Pt3g037920.1 | 3 | - | 41367822 | 41370276 | 2 | 1 | 567 | 189 | 21.58 | 9.30 | Ib |
| PtrWRKY28 | Pt3g037960.1 | 3 | - | 41385601 | 41390746 | 3 | 2 | 1041 | 347 | 37.76 | 6.32 | IId |
| PtrWRKY29 | Pt4g000360.1 | 4 | + | 1281547 | 1285409 | 4 | 3 | 1578 | 526 | 56.76 | 7.73 | Ia |
| PtrWRKY30 | Pt4g000580.1 | 4 | - | 1165631 | 1171631 | 3 | 2 | 876 | 292 | 32.00 | 6.32 | Ib |
| PtrWRKY31 | Pt4g003100.1 | 4 | - | 1909346 | 1911508 | 4 | 3 | 1416 | 472 | 51.75 | 7.61 | IIb |
| PtrWRKY32 | Pt4g004740.1 | 4 | - | 4537358 | 4539993 | 2 | 1 | 582 | 194 | 21.67 | 9.12 | Ib |
| PtrWRKY33 | Pt4g012940.1 | 4 | - | 9434521 | 9437700 | 3 | 2 | 1032 | 344 | 38.48 | 6.75 | Ib |
| PtrWRKY34 | Pt4g014960.1 | 4 | + | 12930390 | 12931700 | 2 | 1 | 489 | 163 | 18.22 | 9.58 | Ib |
| PtrWRKY35 | Pt4g019950.1 | 4 | - | 19870724 | 19872823 | 3 | 2 | 1074 | 358 | 39.51 | 6.34 | IIIa |
| PtrWRKY36 | Pt4g019960.1 | 4 | + | 19866661 | 19868344 | 3 | 2 | 843 | 281 | 31.67 | 7.07 | IIIa |
| PtrWRKY37 | Pt5g004720.1 | 5 | - | 3153830 | 3155215 | 4 | 3 | 960 | 320 | 34.99 | 9.02 | IIc |
| PtrWRKY38 | Pt5g020330.1 | 5 | + | 18817292 | 18819121 | 5 | 4 | 1182 | 394 | 43.91 | 6.20 | IIb |
| PtrWRKY39 | Pt5g025130.1 | 5 | + | 28188102 | 28202874 | 12 | 11 | 3510 | 1170 | 128.90 | 8.43 | Ia |
| PtrWRKY40 | Pt6g002510.1 | 6 | - | 1035787 | 1039057 | 5 | 4 | 1767 | 589 | 63.77 | 6.02 | IIb |
| PtrWRKY41 | Pt6g003530.1 | 6 | - | 371775 | 380324 | 12 | 11 | 2850 | 950 | 106.29 | 6.13 | Ia |
| PtrWRKY42 | Pt6g011450.1 | 6 | + | 7754977 | 7756806 | 3 | 2 | 996 | 332 | 36.84 | 5.50 | IIIa |
| PtrWRKY43 | Pt6g012110.2 | 6 | + | 8291351 | 8296095 | 5 | 4 | 1707 | 569 | 62.58 | 6.48 | Ia |
| PtrWRKY44 | Pt6g016880.1 | 6 | + | 13248183 | 13253514 | 4 | 3 | 1407 | 469 | 51.94 | 8.27 | Ia |
| PtrWRKY45 | Pt7g002860.1 | 7 | - | 2954441 | 2958201 | 3 | 2 | 693 | 231 | 25.91 | 9.10 | Ib |
| PtrWRKY46 | Pt7g005910.1 | 7 | - | 8177323 | 8179704 | 5 | 4 | 969 | 323 | 35.75 | 8.35 | IIa |
| PtrWRKY47 | Pt7g017020.1 | 7 | + | 17890670 | 17893190 | 3 | 2 | 1083 | 361 | 39.66 | 9.32 | IIc |
| PtrWRKY48 | Pt9g001020.1 | 9 | - | 640510 | 642202 | 3 | 2 | 1113 | 371 | 40.74 | 6.05 | Ib |
| PtrWRKY49 | Pt9g018630.1 | 9 | - | 24924385 | 24926321 | 3 | 2 | 1164 | 388 | 42.72 | 5.67 | IIIa |
| PtrWRKY50 | Pt9g019100.1 | 9 | + | 24490253 | 24491907 | 3 | 2 | 1284 | 428 | 47.50 | 4.94 | IId |
| PtrWRKY51 | PtUn004120.1 | UN | + | 4757565 | 4760338 | 3 | 2 | 1080 | 360 | 40.54 | 9.80 | IIc |
