# Supplementary figures and images for: Genome-wide identification and comparative expression profiling of the WRKY transcription factor family in two Citrus species with different Candidatus Liberibacter asiaticus susceptibility
Source: BMC Plant Biol. 2023 Mar 24;23:159. doi: 10.1186/s12870-023-04156-4 (PMC10037894; doi:10.1186/s12870-023-04156-4)

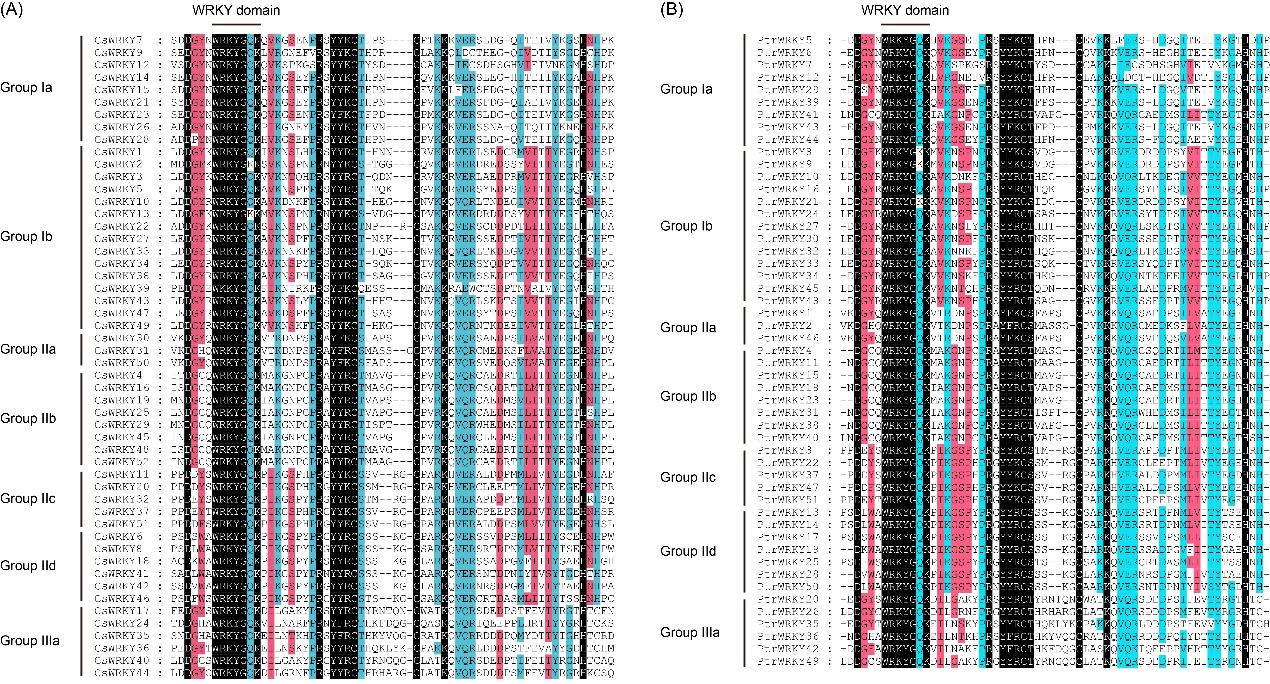


**Additional file 4: Fig. S1. Multiple sequence alignment of WRKY domains in CsWRKYs (A) and PtrWRKYs (B).**

Supplement: Supplementary file 4 — Additional file 4: Figure S1. Multiple sequence alignment of WRKY domains in CsWRKYs and PtrWRKYs [file 12870_2023_4156_MOESM4_ESM.docx]
