# Supplementary material for: Genome-wide identification and comparative expression profiling of the WRKY transcription factor family in two Citrus species with different Candidatus Liberibacter asiaticus susceptibility
Source: BMC Plant Biol. 2023 Mar 24;23:159. doi: 10.1186/s12870-023-04156-4 (PMC10037894; doi:10.1186/s12870-023-04156-4)
Supplement: Supplementary file 5 — Additional file 5: Table S4. Conserved motifs in CsWRKY and PtrWRKY proteins. [file 12870_2023_4156_MOESM5_ESM.docx]

**Additional file 5: Table S4. Conserved motifs in CsWRKY and PtrWRKY proteins**

| **Motif** | **Width** | **E-value** | **Consensus Sequence** |
| --- | --- | --- | --- |
| Motif 1^1^ | 30 | 1.4e-2494 | DILDDGYRWRKYGQKVVKGSPYPRSYYRCT |
| Motif 2 | 41 | 3.7e-2046 | SPGCPVRKQVZRSSEDPSILITTYEGEHNHPLPAAATSMAS |
| Motif 3^1^ | 50 | 6.6e-654 | DGYNWRKYGQKQVKGSEYPRSYYKCTHPNCPVKKKVERSLDGQITEIIYK |
| Motif 4 | 21 | 4.5e-344 | KKKGZKKVREPRVAVQTRSEV |
| Motif 5^2^ | 50 | 1.9e-309 | DDKKVKQELEVLQAELNRVREENERLRKMLNQVTKDYNALQLQLMAJMQK |
| Motif 6 | 29 | 1.2e-198 | LVSAATAAJTADPNFTAALAAAISSIIGG |
| Motif 7 | 29 | 4.1e-149 | SSSSASLSASAPFPTITLDLTHSPNPSSF |
| Motif 8^3^ | 24 | 4.0e-136 | SSGRCHCSKRRKSRVKKVIRVPAI |
| Motif 9 | 29 | 8.1e-118 | TTSAAASMLLSGSSSSADGLSSSGSLAAA |
| Motif 10 | 21 | 1.8e-071 | GEHNHPKPQPTRRSSSSSSMS |
| ^1^Conserved sequences within the WRKY domain; ^2^bZIP motif; ^3^Plant zinc-cluster domain | | | |
