# Supplementary material for: Genome-wide identification and comparative expression profiling of the WRKY transcription factor family in two Citrus species with different Candidatus Liberibacter asiaticus susceptibility
Source: BMC Plant Biol. 2023 Mar 24;23:159. doi: 10.1186/s12870-023-04156-4 (PMC10037894; doi:10.1186/s12870-023-04156-4)
Supplement: Supplementary file 6 — Additional file 6: Table S5. qRT-PCR values of WRKY genes under SA treatment [file 12870_2023_4156_MOESM6_ESM.docx]

**Additional file 6: Table S5. qRT-PCR values of *WRKY* genes under SA treatment**

| **Gene name** | **qPCR fold change** | | | **Gene name** | **qPCR fold change** | | |
| --- | --- | --- | --- | --- | --- | --- | --- |
|  | 0 h | 3 h | 6 h |  | 0 h | 3 h | 6 h |
| *CsWRKY1* | 1.00 | 1.41 | 0.85 | *PtrWRKY1* | 1.00 | 55.11 | 35.57 |
| *CsWRKY2* | 1.00 | 4.98 | 3.53 | *PtrWRKY2* | 1.00 | 148.55 | 92.41 |
| *CsWRKY3* | 1.00 | 1.33 | 0.86 | *PtrWRKY3* | 1.00 | 5.12 | 2.31 |
| *CsWRKY4* | 1.00 | 9.99 | 28.34 | *PtrWRKY4* | 1.00 | 1.87 | 1.13 |
| *CsWRKY5* | 1.00 | 1.33 | 0.96 | *PtrWRKY5* | 1.00 | 1.77 | 1.01 |
| *CsWRKY6* | 1.00 | 4.37 | 2.52 | *PtrWRKY6* | 1.00 | 1.52 | 1.21 |
| *CsWRKY7* | 1.00 | 7.75 | 11.44 | *PtrWRKY7* | 1.00 | 1.97 | 1.60 |
| *CsWRKY8* | 1.00 | 1.56 | 2.08 | *PtrWRKY8* | 1.00 | 12.75 | 9.62 |
| *CsWRKY9* | 1.00 | 1.92 | 1.47 | *PtrWRKY9* | 1.00 | 12.75 | 9.62 |
| *CsWRKY10* | 1.00 | 1.44 | 1.25 | *PtrWRKY10* | 1.00 | 1.81 | 1.51 |
| *CsWRKY11* | 1.00 | 4.08 | 2.81 | *PtrWRKY11* | 1.00 | 4.29 | 3.09 |
| *CsWRKY12* | 1.00 | 1.68 | 1.66 | *PtrWRKY12* | 1.00 | 1.67 | 1.23 |
| *CsWRKY13* | 1.00 | 8.19 | 24.49 | *PtrWRKY13* | 1.00 | 2.57 | 3.57 |
| *CsWRKY14* | 1.00 | 1.88 | 1.81 | *PtrWRKY14* | 1.00 | 4.98 | 4.20 |
| *CsWRKY15* | 1.00 | 1.92 | 1.46 | *PtrWRKY15* | 1.00 | 3.98 | 3.08 |
| *CsWRKY16* | 1.00 | 5.47 | 3.58 | *PtrWRKY16* | 1.00 | 1.81 | 1.72 |
| *CsWRKY17* | 1.00 | 6.76 | 9.50 | *PtrWRKY17* | 1.00 | 3.45 | 0.98 |
| *CsWRKY18* | 1.00 | 0.56 | 0.44 | *PtrWRKY18* | 1.00 | 0.43 | 0.12 |
| *CsWRKY19* | 1.00 | 0.37 | 0.11 | *PtrWRKY19* | 1.00 | 0.67 | 0.47 |
| *CsWRKY20* | 1.00 | 2.73 | 1.58 | *PtrWRKY20* | 1.00 | 3.71 | 2.67 |
| *CsWRKY21* | 1.00 | 2.08 | 1.16 | *PtrWRKY21* | 1.00 | 6.67 | 20.55 |
| *CsWRKY22* | 1.00 | 0.65 | 0.22 | *PtrWRKY22* | 1.00 | 2.73 | 2.23 |
| *CsWRKY23* | 1.00 | 8.37 | 7.72 | *PtrWRKY23* | 1.00 | 4.74 | 3.33 |
| *CsWRKY24* | 1.00 | 259.17 | 92.20 | *PtrWRKY24* | 1.00 | 2.32 | 1.74 |
| *CsWRKY25* | 1.00 | 1.13 | 1.95 | *PtrWRKY25* | 1.00 | 1.08 | 1.11 |
| *CsWRKY26* | 1.00 | 1.52 | 1.48 | *PtrWRKY26* | 1.00 | 7.60 | 5.43 |
| *CsWRKY27* | 1.00 | 1.27 | 0.56 | *PtrWRKY27* | 1.00 | 1.56 | 2.00 |
| *CsWRKY28* | 1.00 | 2.71 | 1.43 | *PtrWRKY28* | 1.00 | 2.07 | 1.05 |
| *CsWRKY29* | 1.00 | 2.08 | 1.89 | *PtrWRKY29* | 1.00 | 2.45 | 1.43 |
| *CsWRKY30* | 1.00 | 94.45 | 60.79 | *PtrWRKY30* | 1.00 | 1.22 | 1.10 |
| *CsWRKY31* | 1.00 | 157.31 | 199.60 | *PtrWRKY31* | 1.00 | 1.63 | 1.27 |
| *CsWRKY32* | 1.00 | 5.54 | 3.38 | *PtrWRKY32* | 1.00 | 3.62 | 1.78 |
| *CsWRKY33* | 1.00 | 4.87 | 4.62 | *PtrWRKY33* | 1.00 | 1.83 | 0.55 |
| *CsWRKY34* | 1.00 | 3.67 | 1.51 | *PtrWRKY34* | 1.00 | 1.67 | 1.32 |
| *CsWRKY35* | 1.00 | 47.21 | 33.52 | *PtrWRKY35* | 1.00 | 1.76 | 1.39 |
| *CsWRKY36* | 1.00 | 1.46 | 1.25 | *PtrWRKY36* | 1.00 | 41.83 | 35.79 |
| *CsWRKY37* | 1.00 | 1.33 | 0.92 | *PtrWRKY37* | 1.00 | 6.10 | 3.26 |
| *CsWRKY38* | 1.00 | 1.70 | 1.05 | *PtrWRKY38* | 1.00 | 0.55 | 0.10 |
| *CsWRKY39* | 1.00 | 7.59 | 4.08 | *PtrWRKY39* | 1.00 | 5.43 | 2.07 |
| *CsWRKY40* | 1.00 | 1.70 | 1.05 | *PtrWRKY40* | 1.00 | 1.56 | 1.88 |
| *CsWRKY41* | 1.00 | 3.02 | 1.89 | *PtrWRKY41* | 1.00 | 2.15 | 1.79 |
| *CsWRKY42* | 1.00 | 2.32 | 1.55 | *PtrWRKY42* | 1.00 | 49.22 | 30.96 |
| *CsWRKY43* | 1.00 | 1.43 | 1.87 | *PtrWRKY43* | 1.00 | 5.16 | 3.09 |
| *CsWRKY44* | 1.00 | 10.30 | 12.18 | *PtrWRKY44* | 1.00 | 1.28 | 1.17 |
| *CsWRKY45* | 1.00 | 0.32 | 0.10 | *PtrWRKY45* | 1.00 | 0.65 | 0.62 |
| *CsWRKY46* | 1.00 | 1.66 | 2.60 | *PtrWRKY46* | 1.00 | 42.30 | 60.47 |
| *CsWRKY47* | 1.00 | 3.62 | 2.97 | *PtrWRKY47* | 1.00 | 2.24 | 1.24 |
| *CsWRKY48* | 1.00 | 5.09 | 4.01 | *PtrWRKY48* | 1.00 | 1.92 | 1.82 |
| *CsWRKY49* | 1.00 | 1.42 | 0.94 | *PtrWRKY49* | 1.00 | 2.90 | 1.39 |
| *CsWRKY50* | 1.00 | 39.57 | 75.83 | *PtrWRKY50* | 1.00 | 1.76 | 1.39 |
| *CsWRKY51* | 1.00 | 3.58 | 2.32 | *PtrWRKY51* | 1.00 | 1.91 | 1.99 |
| *CsWRKY52* | 1.00 | 7.39 | 9.06 |  |  |  |  |
