# Supplementary material for: Genome-wide identification and comparative expression profiling of the WRKY transcription factor family in two Citrus species with different Candidatus Liberibacter asiaticus susceptibility
Source: BMC Plant Biol. 2023 Mar 24;23:159. doi: 10.1186/s12870-023-04156-4 (PMC10037894; doi:10.1186/s12870-023-04156-4)
Supplement: Supplementary file 7 — Additional file 7: Figure S2. The uncropped gel of Fig. 6A-B. [file 12870_2023_4156_MOESM7_ESM.docx]

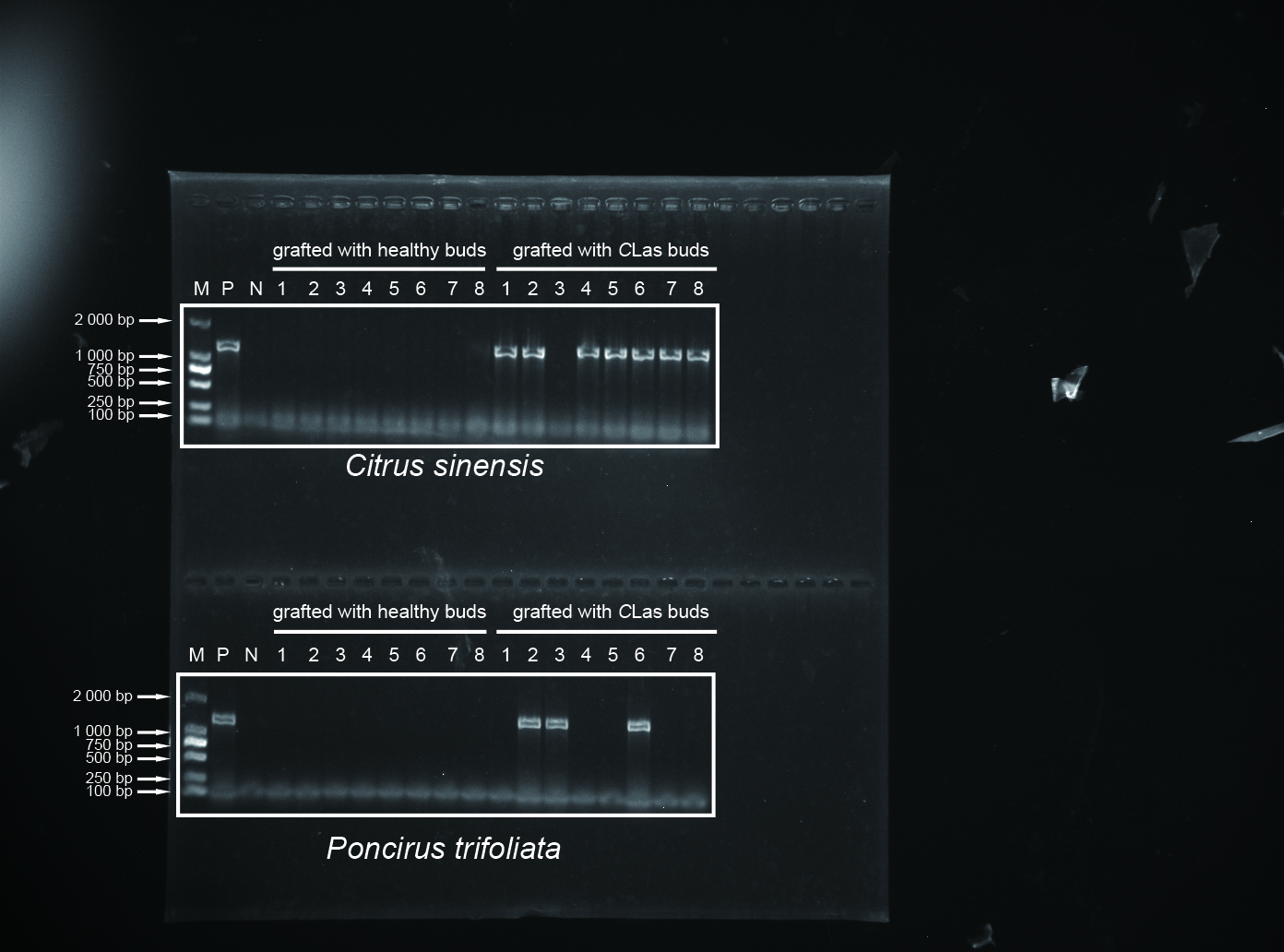


**Additional file 7: Fig. S2. The uncropped gel of Figure 6A-B.**

The white blocks indicate where they were cropped.
