# Supplementary material for: Genome-wide identification and comparative expression profiling of the WRKY transcription factor family in two Citrus species with different Candidatus Liberibacter asiaticus susceptibility
Source: BMC Plant Biol. 2023 Mar 24;23:159. doi: 10.1186/s12870-023-04156-4 (PMC10037894; doi:10.1186/s12870-023-04156-4)
Supplement: Supplementary file 8 — Additional file 8: Table S6. qRT-PCR values of WRKY genes under HLB infection [file 12870_2023_4156_MOESM8_ESM.docx]

**Additional file 8: Table S6. qRT-PCR values of *WRKY* genes under HLB infection**

| **Gene name** | **WT** | **HLB** | **Gene name** | **WT** | **HLB** |
| --- | --- | --- | --- | --- | --- |
| *CsWRKY1* | 1.00 | 1.67 | *PtrWRKY1* | 1.00 | 0.16 |
| *CsWRKY2* | 1.00 | 0.22 | *PtrWRKY2* | 1.00 | 0.45 |
| *CsWRKY3* | 1.00 | 1.67 | *PtrWRKY3* | 1.00 | 5.52 |
| *CsWRKY4* | 1.00 | 17.99 | *PtrWRKY4* | 1.00 | 10.55 |
| *CsWRKY5* | 1.00 | 0.16 | *PtrWRKY5* | 1.00 | 1.00 |
| *CsWRKY6* | 1.00 | 0.19 | *PtrWRKY6* | 1.00 | 0.23 |
| *CsWRKY7* | 1.00 | 0.31 | *PtrWRKY7* | 1.00 | 0.11 |
| *CsWRKY8* | 1.00 | 0.16 | *PtrWRKY8* | 1.00 | 1.22 |
| *CsWRKY9* | 1.00 | 0.14 | *PtrWRKY9* | 1.00 | 1.24 |
| *CsWRKY10* | 1.00 | 1.23 | *PtrWRKY10* | 1.00 | 0.01 |
| *CsWRKY11* | 1.00 | 0.61 | *PtrWRKY11* | 1.00 | 6.33 |
| *CsWRKY12* | 1.00 | 0.23 | *PtrWRKY12* | 1.00 | 0.11 |
| *CsWRKY13* | 1.00 | 0.17 | *PtrWRKY13* | 1.00 | 2.55 |
| *CsWRKY14* | 1.00 | 0.24 | *PtrWRKY14* | 1.00 | 0.05 |
| *CsWRKY15* | 1.00 | 0.95 | *PtrWRKY15* | 1.00 | 5.67 |
| *CsWRKY16* | 1.00 | 8.54 | *PtrWRKY16* | 1.00 | 1.23 |
| *CsWRKY17* | 1.00 | 0.10 | *PtrWRKY17* | 1.00 | 6.12 |
| *CsWRKY18* | 1.00 | 3.69 | *PtrWRKY18* | 1.00 | 0.05 |
| *CsWRKY19* | 1.00 | 4.69 | *PtrWRKY19* | 1.00 | 8.95 |
| *CsWRKY20* | 1.00 | 0.20 | *PtrWRKY20* | 1.00 | 0.44 |
| *CsWRKY21* | 1.00 | 0.20 | *PtrWRKY21* | 1.00 | 12.55 |
| *CsWRKY22* | 1.00 | 0.00 | *PtrWRKY22* | 1.00 | 0.95 |
| *CsWRKY23* | 1.00 | 0.25 | *PtrWRKY23* | 1.00 | 1.32 |
| *CsWRKY24* | 1.00 | 8.11 | *PtrWRKY24* | 1.00 | 6.46 |
| *CsWRKY25* | 1.00 | 0.06 | *PtrWRKY25* | 1.00 | 0.89 |
| *CsWRKY26* | 1.00 | 0.28 | *PtrWRKY26* | 1.00 | 0.59 |
| *CsWRKY27* | 1.00 | 0.45 | *PtrWRKY27* | 1.00 | 1.55 |
| *CsWRKY28* | 1.00 | 0.17 | *PtrWRKY28* | 1.00 | 1.89 |
| *CsWRKY29* | 1.00 | 0.16 | *PtrWRKY29* | 1.00 | 0.22 |
| *CsWRKY30* | 1.00 | 0.10 | *PtrWRKY30* | 1.00 | 0.55 |
| *CsWRKY31* | 1.00 | 0.59 | *PtrWRKY31* | 1.00 | 1.33 |
| *CsWRKY32* | 1.00 | 5.30 | *PtrWRKY32* | 1.00 | 6.22 |
| *CsWRKY33* | 1.00 | 0.46 | *PtrWRKY33* | 1.00 | 0.24 |
| *CsWRKY34* | 1.00 | 1.67 | *PtrWRKY34* | 1.00 | 0.96 |
| *CsWRKY35* | 1.00 | 0.17 | *PtrWRKY35* | 1.00 | 0.56 |
| *CsWRKY36* | 1.00 | 0.46 | *PtrWRKY36* | 1.00 | 0.34 |
| *CsWRKY37* | 1.00 | 0.67 | *PtrWRKY37* | 1.00 | 1.23 |
| *CsWRKY38* | 1.00 | 1.67 | *PtrWRKY38* | 1.00 | 6.16 |
| *CsWRKY39* | 1.00 | 1.90 | *PtrWRKY39* | 1.00 | 3.17 |
| *CsWRKY40* | 1.00 | 0.41 | *PtrWRKY40* | 1.00 | 0.05 |
| *CsWRKY41* | 1.00 | 2.22 | *PtrWRKY41* | 1.00 | 1.23 |
| *CsWRKY42* | 1.00 | 0.94 | *PtrWRKY42* | 1.00 | 11.23 |
| *CsWRKY43* | 1.00 | 1.21 | *PtrWRKY43* | 1.00 | 48.56 |
| *CsWRKY44* | 1.00 | 0.21 | *PtrWRKY44* | 1.00 | 1.23 |
| *CsWRKY45* | 1.00 | 5.96 | *PtrWRKY45* | 1.00 | 1.24 |
| *CsWRKY46* | 1.00 | 1.67 | *PtrWRKY46* | 1.00 | 0.34 |
| *CsWRKY47* | 1.00 | 0.28 | *PtrWRKY47* | 1.00 | 0.22 |
| *CsWRKY48* | 1.00 | 0.60 | *PtrWRKY48* | 1.00 | 1.33 |
| *CsWRKY49* | 1.00 | 0.88 | *PtrWRKY49* | 1.00 | 0.18 |
| *CsWRKY50* | 1.00 | 0.18 | *PtrWRKY50* | 1.00 | 6.12 |
| *CsWRKY51* | 1.00 | 0.33 | *PtrWRKY51* | 1.00 | 0.98 |
| *CsWRKY52* | 1.00 | 0.13 |  |  |  |
