# Supplementary material for: Genome-wide identification and comparative expression profiling of the WRKY transcription factor family in two Citrus species with different Candidatus Liberibacter asiaticus susceptibility
Source: BMC Plant Biol. 2023 Mar 24;23:159. doi: 10.1186/s12870-023-04156-4 (PMC10037894; doi:10.1186/s12870-023-04156-4)
Supplement: Supplementary file 9 — Additional file 9: Table S7. List of stress-responsive cis-acting elements present in 2 kb upstream region of WRKY genes [file 12870_2023_4156_MOESM9_ESM.docx]

**Additional file 9: Table S7. List of stress-responsive *cis*-acting elements present in 2 kb upstream region of *WRKY* genes**

| **Gene name** | ***cis***-element | **Sequence** | **Position** | **Annotation** |
| --- | --- | --- | --- | --- |
| *CsWRKY19* | W box | TTGACC | 1608 | WRKY TF-binding site |
| *CsWRKY19* | MYC | CATTTG | 287 | MYC TF-binding site |
| *CsWRKY19* | MYC | CATGTG | 1627 | MYC TF-binding site |
| *CsWRKY19* | C-repeat/DRE core | GCCGAC | 251 | DREB TF-bindingding site |
| *CsWRKY19* | MYB | CAACAG | 460 | MYB TF-binding site |
| *CsWRKY19* | ABRE | ACGTG | 309 | *cis*-acting element involved in the abscisic acid responsiveness |
| *CsWRKY19* | ABRE | ACGTG | 696 | *cis*-acting element involved in the abscisic acid responsiveness |
| *CsWRKY19* | ABRE | ACGTG | 770 | *cis*-acting element involved in the abscisic acid responsiveness |
| *CsWRKY23* | W box | TTGACC | 1916 | WRKY TF-binding site |
| *CsWRKY23* | C-repeat/DRE core | GCCGAC | 1847 | DREB TF-bindingding site |
| *CsWRKY23* | MYC | CATTTG | 828 | MYC TF-binding site |
| *CsWRKY23* | G-box | TACGTG | 1806 | *cis*-acting regulatory element involved in light responsiveness |
| *CsWRKY23* | ABRE | ACGTG | 685 | *cis*-acting element involved in the abscisic acid responsiveness |
| *CsWRKY23* | ABRE | ACGTG | 1807 | *cis*-acting element involved in the abscisic acid responsiveness |
| *CsWRKY33* | MYC | CAATTG | 298 | MYC TF-binding site |
| *CsWRKY33* | MYC | CATTTG | 329 | MYC TF-binding site |
| *CsWRKY33* | MYC | CATTTG | 983 | MYC TF-binding site |
| *CsWRKY33* | MYC | CATGTG | 1440 | MYC TF-binding site |
| *CsWRKY33* | MYC | CATTTG | 1627 | MYC TF-binding site |
| *CsWRKY33* | W box | TTGACC | 1359 | WRKY TF-binding site |
| *CsWRKY33* | G-box | CACGTG | 415 | *cis*-acting regulatory element involved in light responsiveness |
| *CsWRKY33* | G-box | TACGTG | 549 | *cis*-acting regulatory element involved in light responsiveness |
| *CsWRKY33* | G-box | TACGTG | 1257 | *cis*-acting regulatory element involved in light responsiveness |
| *CsWRKY33* | G-box | TACGTG | 1944 | *cis*-acting regulatory element involved in light responsiveness |
| *CsWRKY47* | MYC | CAATTG | 941 | MYC TF-binding site |
| *CsWRKY47* | W box | TTGACC | 815 | WRKY TF-binding site |
| *CsWRKY47* | MYC | TCTCTTA | 964 | MYC TF-binding site |
| *CsWRKY47* | G-box | TACGTG | 566 | *cis*-acting regulatory element involved in light responsiveness |
| *CsWRKY47* | G-box | CACGTG | 831 | *cis*-acting regulatory element involved in light responsiveness |
| *CsWRKY47* | MYB | TAACCA | 218 | MYB TF-binding site |
| *CsWRKY52* | MYC | CATTTG | 347 | MYC TF-binding site |
| *CsWRKY52* | MYC | CATTTG | 1414 | MYC TF-binding site |
| *CsWRKY52* | ABRE | ACGTG | 1218 | *cis*-acting element involved in the abscisic acid responsiveness |
| *CsWRKY52* | ABRE | ACGTG | 1983 | *cis*-acting element involved in the abscisic acid responsiveness |
| *CsWRKY52* | MYB | CAACAG | 244 | MYB TF-binding site |
| *CsWRKY52* | MYB | TAACCA | 1211 | MYB TF-binding site |
| *CsWRKY6* | MYB | CAACCA | 672 | MYB TF-binding site |
| *CsWRKY6* | MYB | TAACCA | 1737 | MYB TF-binding site |
| *CsWRKY6* | MYC | CAATTG | 191 | MYC TF-binding site |
| *CsWRKY6* | MYC | CATTTG | 976 | MYC TF-binding site |
| *CsWRKY6* | MYB | TAACTG | 445 | MYB TF-binding site |
| *CsWRKY7* | MYB | CAACCA | 1229 | MYB TF-binding site |
| *CsWRKY7* | MYB | CAACCA | 1561 | MYB TF-binding site |
| *CsWRKY7* | G-box | TACGTG | 1982 | *cis*-acting regulatory element involved in light responsiveness |
| *CsWRKY7* | MYC | CAATTG | 73 | MYC TF-binding site |
| *CsWRKY7* | MYC | CAATTG | 533 | MYC TF-binding site |
| *CsWRKY7* | MYC | CATGTG | 1323 | MYC TF-binding site |
| *CsWRKY7* | MYC | CATGTG | 1813 | MYC TF-binding site |
| *CsWRKY7* | MYC | CATGTG | 1874 | MYC TF-binding site |
| *CsWRKY7* | W box | TTGACC | 1057 | WRKY TF-binding site |
| *PtrWRKY11* | MYC | CAATTG | 42 | MYC TF-binding site |
| *PtrWRKY11* | MYC | CAATTG | 733 | MYC TF-binding site |
| *PtrWRKY11* | W box | TTGACC | 606 | WRKY TF-binding site |
| *PtrWRKY11* | MYC | TCTCTTA | 756 | MYC TF-binding site |
| *PtrWRKY11* | G-box | TACGTG | 357 | *cis*-acting regulatory element involved in light responsiveness |
| *PtrWRKY11* | G-box | CACGTG | 622 | *cis*-acting regulatory element involved in light responsiveness |
| *PtrWRKY11* | MYB | CAACCA | 1738 | MYB TF-binding site |
| *PtrWRKY17* | MYB | CAACCA | 316 | MYB TF-binding site |
| *PtrWRKY17* | MYB | TAACCA | 1391 | MYB TF-binding site |
| *PtrWRKY17* | MYC | CATTTG | 638 | MYC TF-binding site |
| *PtrWRKY17* | MYC | CATTTG | 1726 | MYC TF-binding site |
| *PtrWRKY18* | ABRE | ACGTG | 612 | *cis*-acting element involved in the abscisic acid responsiveness |
| *PtrWRKY18* | ABRE | ACGTG | 803 | *cis*-acting element involved in the abscisic acid responsiveness |
| *PtrWRKY18* | G-box | TACGTG | 230 | *cis*-acting regulatory element involved in light responsiveness |
| *PtrWRKY18* | MYC | CATGTG | 1627 | MYC TF-binding site |
| *PtrWRKY18* | W box | TTGACC | 1600 | WRKY TF-binding site |
| *PtrWRKY24* | ABRE | ACGTG | 1545 | *cis*-acting element involved in the abscisic acid responsiveness |
| *PtrWRKY24* | MYB | TAACCA | 701 | MYB TF-binding site |
| *PtrWRKY24* | G-box | CACGTG | 707 | *cis*-acting regulatory element involved in light responsiveness |
| *PtrWRKY24* | MYC | CATTTG | 912 | MYC TF-binding site |
| *PtrWRKY24* | MYC | CATGTG | 1656 | MYC TF-binding site |
| *PtrWRKY24* | MYC | CATTTG | 1749 | MYC TF-binding site |
| *PtrWRKY24* | W box | TTGACC | 1716 | WRKY TF-binding site |
| *PtrWRKY24* | MYB | CAACTG | 1978 | MYB TF-binding site |
| *PtrWRKY32* | MYB | CAACAG | 284 | MYB TF-binding site |
| *PtrWRKY32* | ABRE | CACGTG | 214 | *cis*-acting element involved in the abscisic acid responsiveness |
| *PtrWRKY32* | ABRE | ACGTG | 349 | *cis*-acting element involved in the abscisic acid responsiveness |
| *PtrWRKY32* | ABRE | ACGTG | 1059 | *cis*-acting element involved in the abscisic acid responsiveness |
| *PtrWRKY32* | ABRE | ACGTG | 1748 | *cis*-acting element involved in the abscisic acid responsiveness |
| *PtrWRKY32* | MYB | TAACTG | 1359 | MYB TF-binding site |
| *PtrWRKY32* | W box | TTGACC | 1161 | WRKY TF-binding site |
| *PtrWRKY32* | MYC | CATTTG | 128 | MYC TF-binding site |
| *PtrWRKY32* | MYC | CATGTG | 1242 | MYC TF-binding site |
| *PtrWRKY32* | MYC | CATTTG | 1426 | MYC TF-binding site |
| *PtrWRKY39* | MYB | CAACCA | 1920 | MYB TF-binding site |
| *PtrWRKY39* | MYB | CAACTG | 381 | MYB TF-binding site |
| *PtrWRKY39* | MYB | TAACTG | 412 | MYB TF-binding site |
| *PtrWRKY39* | MYB | CAACTG | 471 | MYB TF-binding site |
| *PtrWRKY39* | MYB | CAACTG | 732 | MYB TF-binding site |
| *PtrWRKY39* | MYB | CAACTG | 1708 | MYB TF-binding site |
| *PtrWRKY39* | W box | TTGACC | 1811 | WRKY TF-binding site |
| *PtrWRKY43* | W box | TTGACC | 1704 | WRKY TF-binding site |
| *PtrWRKY43* | C-repeat/DRE core | GCCGAC | 1636 | DREB TF-bindingding site |
| *PtrWRKY43* | MYC | CATTTG | 607 | MYC TF-binding site |
| *PtrWRKY43* | MYC | CATTTG | 1985 | MYC TF-binding site |
| *PtrWRKY43* | G-box | TACGTG | 1591 | *cis*-acting regulatory element involved in light responsiveness |
| *PtrWRKY43* | ABRE | ACGTG | 463 | *cis*-acting element involved in the abscisic acid responsiveness |
